# Optimization of Huang-Lian-Jie-Du-Decoction for Ischemic Stroke Treatment and Mechanistic Study by Metabolomic Profiling and Network Analysis

**DOI:** 10.3389/fphar.2017.00165

**Published:** 2017-03-28

**Authors:** Qian Zhang, Junsong Wang, Shanting Liao, Pei Li, Dingqiao Xu, Yan Lv, Minghua Yang, Lingyi Kong

**Affiliations:** ^1^State Key Laboratory of Natural Medicines, Department of Natural Medicinal Chemistry, China Pharmaceutical UniversityNanjing, China; ^2^Center for Molecular Metabolism, Nanjing University of Science and TechnologyNanjing, China

**Keywords:** metabolomics, ischemic stroke, correlation network, orthogonal experimental design, amino acids, middle cerebral artery occlusion, Huang-Lian-Jie-Du-Decoction

## Abstract

Optimal drug proportions and mechanism deciphering of multicomponent drugs are critical for developing novel therapies to cope with complex diseases, such as stroke. In the present study, orthogonal experimental design was applied to explore the optimal proportion of the four component herbs in Huang-Lian-Jie-Du-Decoction (HLJDD) on the treatment of ischemic stroke. The treatment efficacies and mechanisms were assessed using global and amino acids (AAs) targeted metabolomics, as well as correlation network analysis. The global NMR metabolomics results revealed that AAs metabolism was significantly perturbed in middle cerebral artery occlusion rats. The levels of 23 endogenous AAs were then subjected to HPLC-QTOF-MS/MS analysis. These results complemented with neurobehavioral evaluations, cerebral infarct assessments, biochemical evaluations, histological inspections and immunohistochemistry observations strongly demonstrated that HLJDD with optimal proportion of 6 (*Rhizoma coptidis*): 4 (*Radix scutellariae*): 1 (*Cortex phellodendr*): 3 (*Fructus Gardeniae*) had the best efficacy on ischemic stroke, which could be ascribed to its modulation on AA metabolism. This integrated metabolomics approach showed the potential and applicable in deciphering the complex mechanisms of traditional Chinese medicine formulae on the treatment of complicated diseases, which provided new means to assess the treatment effects of herb combinations and to further development of drugs or therapies based on these formulae.

## Introduction

Stroke is one of the leading causes of death and adult disability worldwide, and ischemic stroke accounts for 87% of all forms of stroke patients (Strong et al., 2007; Lloyd-Jones et al., 2010). Unfortunately, no effective and generally accepted treatment for ischemic stroke is available now due to its complexity: multiple events occurred in ischemic stroke, such as excitotoxic amino acids (AAs), inflammation, oxidative damage, ionic imbalances, apoptosis, angiogenesis and neuroethology injury. Therefore, it is a huge challenge to rectify these abnormalities in clinic, especially with a single-target drug or monotherapy. Increasing evidences in treating complex illness such as ischemic stroke demonstrate that treatment regimens containing multiple drugs with distinct but related mechanisms can usually amplify the therapeutic efficacies of each agent, leading to maximal therapeutic efficacy with minimal adverse effects. Therefore, combination therapy has been a rational and promising choice (Amarenco, 2006; Kernan et al., 2010).

Actually, combination therapies have been advocated by prescriptions in the form of formulae in traditional Chinese medicine (TCM) for more than 2,500 years to prevent and cure diseases based on clinical practice (Wang et al., 2008). There is no doubt that, at least in some formulae, multiple components could hit multiple targets simultaneously and exert synergistic therapeutic efficacies. It is thus important to elucidate the complex action mechanism of TCM formulae to improve its clinical efficacy and safety, and for further drug or therapy development (Wang et al., 2008). Understanding optimal drug proportions and synergistic mechanisms of multicomponent drugs are critical for developing novel strategies to cope with complex diseases, such as stroke.

The double complexity of ischemic stroke and the components of the formula created a far more difficult situation to assess the efficacy of the formula, needless to say, to clarify the combinatorial rules of herbal formula. Therefore, it’s urgent to employ new methods to elucidate the complex action mechanisms of TCM formulae, which is essential for further development of drugs or therapies based on these formulae. Metabolomics has been used as a versatile tool for the discovery of disease-related biomarkers and in assessment of the therapeutic effects of drug (Shah et al., 2012; Fan et al., 2016; Ma et al., 2016). The holistic view of metabolomics is similar to the theory and practice of TCM on the treatment of disease. Therefore, it is reasonable to apply metabolomics technology to explore the effects of drug combination and the underlying mechanisms of TCM formula.

Huang-Lian-Jie-Du-Decoction (HLJDD, Oren-gedoku-to in Japanese), consisting of *Rhizoma coptidis* (Rc), *Radix scutellariae* (Rs), *Cortex phellodendr* (Cp), and *Fructus Gardeniae* (Fg), is a famous TCM formula for heat clearance and detoxification, which has been used to treat ischemic stroke in the clinical practice of TCM in China and other Asian countries (Kawashima et al., 1988; Kondo et al., 2000; Itoh, 2001). HLJDD acted as a potential neuroprotective agent and offered protection on ischemic injury (Hwang et al., 2002; Wang et al., 2014; Zhang et al., 2015). However, the optimal proportions and combinational mechanisms of HLJDD on the treatment of ischemic stroke remain unidentified.

In the present study, a middle cerebral artery occlusion (MCAO) rat model was used to mimic ischemic stroke, and orthogonal experimental design was proposed to evaluate the optimal proportions of the four component herbs in HLJDD. Furthermore, proton nuclear magnetic resonance (^1^H NMR)-based global metabolomics and high-performance liquid chromatography time-of-flight mass spectrometry (HPLC-QTOF-MS) targeted metabolomics, as well as metabolic correlation networks were used to assess the combinational mechanisms.

## Materials and Methods

### Materials and the Preparation of Formulae 1–9

Sodium 3-trimethylsilyl-propionic acid (TSP) was purchased from Sigma (St. Louis, MO, USA). Deuterium oxide (D_2_O, 99.9%) was bought from Sea Sky Bio Technology Co. Ltd (Beijing, China). Chloral hydrate was obtained from Sinopharm Chemical Reagent Co. Ltd (Shanghai, China). Phenylisothiocyanate (PITC) was purchased from Dalian Meilun Biotech Co., Ltd. (Dalian, China). AAs standard solution was supplied from Sigma (Taufkirchen, Germany, mix of 17 AAs in hydrogen chloride solution, 2.5 mmol/L in 0.1 mol/L HCl, except for l-cystine at 1.25 mmol/L). The standard consists of 17 AAs that are l-Aspartic acid (Asp), l-Serine (Ser), l-Glutamate (Glu), l-Glycine (Gly), l-Histidine (His), l-Arginine (Arg), l-Threonine (Thr), l-Alanine (Ala), l-Proline (Pro), l-Cystine (Cys), l-Tyrosine (Tyr), l-Valine (Val), l-Methionine (Met), l-Lysine (Lys), l-Isoleucine (Ile), l-Leucine (Leu), and l-Phenylalanine (Phe). Taurine (Tau), l-Glutamine (Gln), l-Ornithine (Orn), l-Tryptophan (Trp), γ-AminoButyric Acid (GABA) and DL-2-aminobutyric acid (2-ABA), and internal standard l-Norleucine (IS) were obtained from Aladdin (Shanghai, China). Acetonitrile (LC-MS grade), ammonium acetate and acetic acid (LC-MS grade) were bought from ROE Scientific Inc. (Newark, NJ, USA). The distilled water (Watsons Water, HK) used in the experiment was filtered through 0.22 μm nylon membrane prior to use.

*Rhizoma Coptidis* (*Coptis chinensis* Franch, Ranunculaceae), *Radix Scutellariae* (*Scutellaria baicalensis* Georgi, Labiatae), *Cortex Phellodendri* (*Phellodendron chinensis* Schneid, Rutaceae) and *Fructus Gardeniae* (*Gardenia jasminoides* Ellis, Rubiaceae) were provided by Jiangsu Medicine Company (Nanjing, China, Drug GMP certificate: SUJ0623. Drug Manufacturing Certificate: SUY20110051), and authenticated by Professor Mian Zhang, Department of Medicinal Plants, China Pharmaceutical University, Nanjing, China. Formulae 1–9 reaching a total weight of 1.0 kg (the weight ratios were list in Supplementary Table S1), were extracted with water (1:10, w/v) under reflux for three times, 1 h each. The extract solutions were combined and freeze-drying in vacuum to afford extracts of formulae 1–9 (the yields were list in Supplementary Table S1), which were dissolved in 0.5% CMC-Na (carboxymethyl cellulose sodium salt) to the final concentration of 5.0 g/ml (equivalent to dry weight of raw materials) before intragastrical (i.g.) administration.

### Animal Handling Procedure and Drug Administration

Male Sprague-Dawley rats weighing 250 ± 20 g were obtained from the Experimental Animal Center of Yangzhou University (Yangzhou, China). All animals were housed in a well-ventilated room (five rats in one cage) at a constant room temperature (25 ± 2°C) and controlled humidity (50 ± 10%), with a 12 h dark-light cycle and free access to chow and water. The rats were allowed to acclimatize for 7 days before the experiments. All procedures for animal care and use were in accordance with the National Institute of Health (NIH) guidelines for the Care and Use of Laboratory Animals, and approved by the Institutional Animal Care and Use Committee of China Pharmaceutical University [license number: SYXK (Su) 2016-0011].

The MCAO model in rats was performed as previously described (Wang et al., 2014). Briefly, animals were firstly anesthetized with chloral hydrate (3.0%, 350 mg/kg, i.p.). The right common carotid artery (CCA), the right external carotid artery (ECA), and the right internal carotid artery (ICA) were exposed and isolated from connective tissues. A poly-l-lysine coated nylon monofilament (0.26 mm, 2636-A3, Beijing Cinontech Co. Ltd., Beijing, China) with the tip heat rounded diameter of 0.36 ± 0.02 mm) was inserted into the ICA through the ECA until its tip was lodged in the anterior cerebral artery (ACA), a distance of 18–20 mm from the carotid bifurcation according to the weight of the animal, to obstruct the blood flow into the middle cerebral artery (MCA), thus achieving cerebral ischemia. After 2 h sustained ischemia, reperfusion was performed by the withdrawal of the inserted filament. Sham-operated rats received the same surgical procedures except that the arteries were not occluded. All surgical operations were done in a sterile environment. Rectal temperature of the animal was monitored by a thermistor and maintained at 37.0 ± 0.5°C with a thermostatically controlled heating pad (ALCBIO, Shanghai, People’s Republic of China) during surgery and ischemia.

In addition to the sham operation group (NC, *n* = 20) and MCAO model group (M, *n* = 40), rats were randomly assigned to nine treatment groups (F1–F9), treated by different combination of HLJDD component herbs according to an orthogonal experimental design (*n* = 40/group, **Table 1**). As shown in Supplementary Figures S1–S6, 37 components in F1–F9 extracts were identified, mainly involving alkaloids, flavonoids, terpenoids, phenols, iridoids and their glycosides (Supplementary Tables S2–S6). Formulae 1–9 were dissolved in 0.5% CMC-Na (carboxymethyl cellulose sodium salt) and intragastrically (i.g.) administered to rats (5 g/kg, weight ratio between crude drug and rat for each administration) once per day for 10 consecutive days, while the sham and MCAO groups were i.g. administrated with an equivalent amount of 0.5% CMC-Na. Rats in the MCAO group and nine treatment groups (F1–F9) underwent MCAO surgery, while rats in the sham group only received arteries separation without filament insertion. The rats were fasted for 12 h before the operation of the MCAO model but were allowed free access to water. The flow diagram of the present experimental design was shown in Supplementary Figure S7.

**Table 1:** The orthogonal experimental design L_9_(3^4^) of HLJDD.

groups	Rhizoma Coptidis	Radix Scutellariae	Cortex Phellodendri	Fructus Gardeniae
F1	3	2	2	3
F2	6	2	2	3
F3	3	4	4	6
F4	3	4	2	12
F5	3	2	8	6
F6	3	8	4	3
F7	12	4	8	3
F8	6	1	2	6
F9	6	4	1	3

### Orthogonal Experimental Design

To explore the optimal proportions of HLJDD, orthogonal experimental design was used. For four parameters at three levels each, the traditional full factorial design would require 3^4^ or 81 experiments. However, current design (Taguchi L9 orthogonal array) required only nine experiments (Yang and Tarng, 1998). It uses an orthogonal table to organize the factors and levels of each factor. The table is denoted as L_9_ (4^3^), representing an orthogonal design of nine experiments, involving four factors at three levels (Supplementary Table S7). The details of the orthogonal design of HLJDD were shown in **Table 1**, the four component herbs of HLJDD were reformulated to eight HLJDD variants F2–F9.

### Neurological Defects and Infarct Volume Measurements

At 24 h after reperfusion, each animal’s neurological function was evaluated. The neurological scores were recorded by an investigator who was blinded to the experimental groups according to Longa’s five-point scale (Longa et al., 1989): 0, no neurological deficit; 1, failure to extend right forelimb; 2, circling to the contralateral side; 3, falling to the contralateral side at rest; and 4, no spontaneous motor activity.

Cerebral infarct volumes were evaluated by 2,3,5-triphenyltetrazolium chloride (TTC, Sigma) staining method (Bederson et al., 1986). The brains were sectioned into six 2-mm-thick coronal slices, stained with 1% TTC, and incubated at 37°C for 30 min in the dark, and then fixed with 10% buffered formalin overnight. Normal tissue was stained rose red, and the infarct tissue was stained white. Slices stained with TTC were photographed, and analyzed using image analysis software (Image-Pro Plus 6.0). Tests were conducted by an observer blinded to the treatment group. To correct infarct volume (*V*_i_) for brain edema, the percentage of the infarction volumes (*I*%) were obtained using following formula:

I% = (Vc−Vi)/Vc × 100%

*V*c = volume of intact contralateral (left) hemisphere

*V*i = volume of intact regions of the ipsilateral (right) hemisphere (Zhang et al., 2006).

### Biochemistry Evaluation

After 24 h of reperfusion, rats were sacrificed and brains were removed. The homogenates extracted from the ischemic brain hemispheres were analyzed for biochemical parameters. The levels of oxidative stress-related biological components, including nitric oxide (NO), malondialdehyde (MDA), superoxide dismutase (SOD), glutathione (GSH), glutathione peroxidase (GSH-PX), and catalase (CAT) were measured using commercially available kits (Nanjing Jiancheng Biotech Inc., China). People who analyzed the subsequent data did not know this assignment.

### Histopathology and Immunohistochemical Examinations

The brain tissues were immersed in 10% neutral buffered formaldehyde for 24 h, embedded in paraffin, and sliced into 5 μm thickness. The sliced sections were stained with haematoxylin and eosin (H&E), and examined by light microscopy (×200 magnification, Olympus DX45). The histopathology results were evaluated by Prof. Ning Su (Southeast University, Nanjing, China) who was blinded to the experiments.

For immunohistochemical examination, formalin-fixed, paraffin embedded brain tissue sections were used and the activities of glial fibrillary acidic protein (GFAP) and vascular endothelial growth factor (VEGF) were evaluated by Goodbio Technology Co., Ltd. (Nanjing, China). The staining was photographed under light microscopy, and analyzed by a researcher who was blinded to the experimental treatment groups using image analysis software (Image-Pro Plus 6.0).

### Global Metabolomics Study by NMR

#### Sample Preparation and ^1^H NMR Analysis

Cerebrum samples were homogenized and extracted according to our previously published procedures (Wei et al., 2015). Frozen ischemic hemisphere of brains (200–300 mg) were homogenized in ice-cold solvent (50% acetonitrile/H_2_O, v/v, 5 ml/g tissue), vortexed and then centrifuged at 12,000 *g* for 10 min at 4°C. The supernatant was collected and concentrated under a stream of nitrogen and lyophilized. Dried cerebrum extracts were reconstituted in 600 μL D_2_O phosphate buffer (0.2 mol L^-1^ Na_2_HPO_4_ and 0.2 mol L^-1^ NaH_2_PO_4_, pH 7.4, containing 0.05% TSP). TSP acted as a chemical shift reference (δ 0.0), D_2_O provided a lock signal and phosphate buffer was added to minimize NMR shift variation due to the pH discrepancy. After vortexing and centrifugation at 12,000 *g* for 10 min at 4°C to remove insoluble material, the transparent supernatant solution was pipetted into 5 mm NMR tube for NMR analysis.

^1^H NMR spectra of the samples were acquired at 298 K on a Bruker Avance 500 MHz spectrometer (Bruker GmbH, Karlsruhe, Germany). For each cerebrum tissue sample, a nuclear overhauser effect spectroscopy (NOESYPR) pulse sequence (relaxation delay-90°-μs-90°-tm-90°-acquire-FID) was used to attenuate the residual water signal. ^1^H NMR spectra were measured with 128 scans into 32,768 (32 K) data points over a spectral width of 10,000 Hz. Prior to Fourier transformation, an exponential window function with a line broadening of 0.5 Hz was used for the free induction decays (FIDs).

#### Spectral Pre-processing

The spectra for all samples were manually phased and baseline corrected and referenced to TSP at 0.0 ppm, using Bruker Topspin 3.0 software (Bruker GmbH, Karlsruhe, Germany). The ^1^H NMR spectra were automatically exported to ASCII files using MestReNova (Version 8.0.1, Mestrelab Research SL), which were then imported into “R”^1^ and aligned with an in-house developed R-script to further reduce phase and baseline distortions. The one-dimensional (1D) spectra were converted to an appropriate format for statistical analysis by automatically segmenting each spectrum into 0.015-ppm integrated spectral regions (buckets) between 0.2 and 10 ppm. The region of the residual water and affected signals (4.65–5.25 ppm) was removed. To account for different dilutions of samples, all binned spectra were probability quotient normalized and then mean-centered before further multivariate analysis.

#### Data Analysis

The mean-centered and Pareto-scaled NMR data were analyzed by principal component analysis (PCA) and orthogonal partial least-squares discriminant analysis (OPLS-DA). PCA is an exploratory unsupervised method to maximize the separation by providing model-free approaches for determining the latent or intrinsic information in the dataset. However, no clustering was observed when variables were not selected. OPLS-DA determined PLS components that are orthogonal to the grouping and was used to concentrate group discrimination into the first component with remaining unrelated variations contained in subsequent components. The OPLS-DA model was validated using a repeated two fold cross-validation (2CV) and permutation test (2000 times) (Westerhuis et al., 2008) to reduce the chance separation of the two categories and give confidence to the predictive capacity of the models. The parameters *R*^2^ and *Q*^2^ reflected the goodness of fitness and the predictive ability of the models, respectively. The classification performance was evaluated by analyzing the area under the receiver operating characteristic curve (AUROC) and misclassification rate of established models based on permutation test. The fold change values of metabolites between groups were calculated by the ratios of integral non-overlapping areas of each metabolite. The Benjamini and Hochberg (1995) method was used to adjust the *p*-values for controlling the false discovery rate in multiple comparisons using scripts written in R language^2^. The fold change values of metabolites and associated *p*-values adjusted by BH methods in different group comparisons were visualized in fold change plots (Supplementary Figure S8).

#### Targeted Metabolomics Study of Amino Acids (AAs) by HPLC-QTOF-MS

In the targeted analysis, AAs were extracted from the brain tissues and the developed method relies on derivatization of AAs to AA butyl esters using PITC according to published methods with modifications (Robert et al., 1984; Senden et al., 1992; Zheng et al., 2015). Briefly, 100 μL of brain homogenate or AAs standard was mixed with 10 μL of internal standard l-Norleucine solution in a microcentrifuge tube. Then, 100 μL of 0.1 mol/L PITC-ACN solution and 100 μL of 1.0 mol/L trimethylamine-ACN solution were added and vortexed for 2 min. After incubating at room temperature for 60 min, 400 μL of n-hexane was added, after shaking for 1 min, standing for 10 min, the lower layer was transferred into another microcentrifuge tube, and then subjected to HPLC-QTOF-MS/MS analysis.

The HPLC analysis of the selected AAs was carried out using an Agilent 1290 HPLC system (Agilent Technologies, Santa Clara, CA, USA). The chromatography separation was performed on an Agilent ZORBAX Eclipse Plus C18 column (2.1 × 100 mm I.D., 3.5 μm particle size) with a solvent flow rate of 1 ml/min at 40°C. The sample injection volume was set at 5 μl. The mobile phase A was composed of 0.01 mol/L sodium acetate solution (pH 6.5) and acetonitrile in a ratio 93:7, and the mobile phase B was water and acetonitrile in a ratio of 20:80. The solvent gradient adopted was as follows: 0–12% B at 0–5 min, 12–15% B at 5–5.1 min, 15–19% B at 5.1–6.1 min, 19–50% B at 6.1–6.2 min, 50–70% B at 6.2–7 min, 70–100% B at 7–9 min, 100% B at 9–11.8 min, 100–0% B at 11.8–13 min, 0% B at 13–20 min. A 5-min post-run time back to the initial mobile phase composition was used after each analysis.

The mass spectrometric data was collected using a tandem quadrupole time-of-flight mass spectrometer (Agilent Technologies, Santa Clara, CA, USA) equipped with an electrospray interface. Electrospray ionization (ESI) was used as the ionization source, and the analysis was carried out in negative mode. The conditions of the ESI source were as follows: drying gas (N2) flow rate, 8.0 L/min; drying gas temperature, 300°C; nebulizer, 241 kPa (35 psig); capillary voltage, 3500 V; fragmentor, 150 V; skimmer voltage, 65 V. All the operations, acquisition and analysis of data were made by Agilent Mass Hunter workstation software version B.04.00 (Agilent Technologies, MA, USA).

#### Metabolites Identified in ^1^H NMR Spectra of Cerebrum Tissues

Representative 500 MHz ^1^H NMR spectra of cerebrum samples from the sham, the MCAO, the F1-treated rats were shown in Supplementary Figure S9 with the assignment of metabolites. The signals were assigned by querying publicly accessible metabolomics databases, such as MMCD^3^ and HMDB^4^, and aided by Chenomx NMR suite 7.5 (Chenomx Inc., Edmonton, AB, Canada). The detailed information of the metabolites was listed in Supplementary Table S8.

#### Method Validation of LC-QTOF-MS/MS Analysis

The LC-QTOF-MS/MS analytical method was developed and validated for simultaneous quantification of 23 AAs in rat brain tissues. The method was validated by determining the linearity, accuracy, precision, recovery, matrix effect, and stability. Calibration was performed using a least-squares linear regression of the peak area ratios of the AAs to the IS versus the respective standard concentration. The low limit of quantification (LLOQ) was obtained at the concentration that provided signal-to-noise ratios larger than 10. The intra-day and inter-day accuracy and precision were evaluated by determination at three different concentrations of quality-control (QC) samples. The intra-day precision was assessed by analysis of AAs in QC samples on the same day. Inter-day precision was evaluated by repeated analysis of AAs in QC samples over three consecutive days. The recovery was assessed by comparing the peak area of QC samples extracted from biological matrix with those pure standards at the same concentration levels without extraction. The matrix effect was evaluated by comparing the peak areas of post-extraction blank plasma samples spiked with AAs to those of the neat standards at the same concentration. The ratios ranged from 85 to 115% were considered as no ionization suppression or enhancement in this method. The stability of AAs in rat brain tissues was assessed by analyzing the three levels of QC samples under various conditions, including at room temperature for 4 h (short-term stability), stored at -80°C for 2 weeks (long term stability), after three freeze/thaw cycles (-80 to 25°C), and at 4°C for 24 h in autosampler vials (post-preparative stability). The samples were considered stable if assay values were within the acceptable limits of accuracy (±15% RE) and precision (≤15% RSD).

#### Correlation Network Analysis

Metabolic correlation networks were performed using the R-package igraph software. In the networks, the nodes represent the metabolites, and the lines between the nodes indicate the biological relationships between the two corresponding metabolites. The solid lines between the molecules indicate a correlation between the molecules, the line colors of red and blue display positive and negative relationships, respectively. Metabolites of similar structures were connected by the dotted lines, indicating a possible biochemical reaction between the molecules.

This improved correlation networks could present both the Pearson correlation coefficients among levels of metabolites and their structure similarity. Common Pearson correlation networks only visualized the correlations of metabolites in different status. However, such correlations were not causation relationships. The addition of structure similarity information enriched the networks since that the substrates and products in nearly all the biochemical reactions should be similar. In this context, the high correlation between metabolites with great structure resemblance might reflect a theoretically possible biochemical reaction between them and thus causative effects.

#### Statistical Analysis

Assays were conducted at least three times unless otherwise stated. All data, except for mortality were expressed as the means ± standard deviation (SD). The data of model groups was compared with that of sham groups and the data of the other groups was compared with the model groups, respectively. Statistical significance was performed using Student’s two-tailed *t*-test for comparison between two groups and one-way analysis of variance (ANOVA) followed by Tukey’s multiple comparison test when the data involved three or more groups. A *p*-value less than 0.05 was considered to be statistically significant.

## Results

### The Improvement of Neurological Defects by Formulae 1–9

**FIGURE 1:**
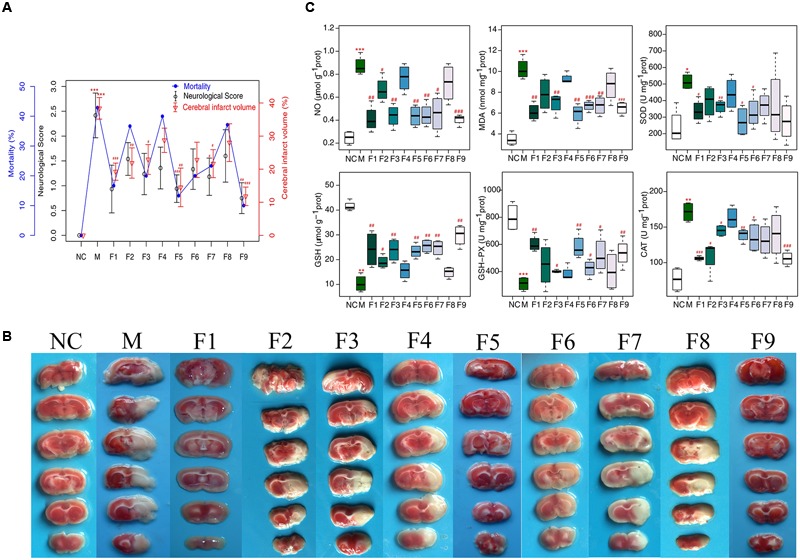
**Mortality, neurological deficits, and infarct volume. (A)** Mortality, neurobehavioral scores and infarct volume examinations. **(B)** TTC staining of brains (*n* = 6). **(C)** Boxplots for brain tissue levels of NO, MDA, SOD, GSH, GSH-Px, and CAT in each group. At the bottom of each box, the lines drew in the box and at the top of the box represent the 1st, 2nd, and 3rd quartiles, respectively. The whiskers extended to ±1.5 times the interquartile range (from the 1st to 3rd quartile). All data are expressed as mean ±SD, *n* = 6. ^∗^*p* < 0.05, ^∗∗^*p* < 0.01, and ^∗∗∗^*p* < 0.001 MCAO group vs. sham group;^#^*p* < 0.05, ^##^*p* < 0.01, and ^###^*p* < 0.001 Treatment groups vs. MCAO group.

At 24 h after reperfusion, as shown in **Figure 1A**, compared to the sham group, the mortality and the neurological score in the MCAO group increased significantly, indicating that MCAO was successfully induced. While in F1, F5, and F9 treated rats, the mortalities and the neurological scores were significantly reduced compared to MCAO rats. According to the quantitative analysis of the infarct volumes and TTC staining of cerebral slices (**Figures 1A,B**), ischemia/reperfusion (I/R) significantly increased the cerebral infarct volume (37.85 ± 2.17%), which was significantly attenuated by F1, F5, and F9 treatments.

### Biochemical Analysis

In our experiment, the levels of oxidative stress-related biologicals were measured (**Figure 1C**). Compared with the sham group, NO and MDA were significantly increased in the I/R group and were decreased markedly in the F1, F5, and F9 groups. The activities of the antioxidases SOD, CAT, and GSH-Px were apparently inhibited in the MCAO group compared with the sham group, which were greatly augmented by treatments with F1, F5, and F9. I/R produced a notable reduction in the quantity of GSH in the MCAO group, which was reversed by F1, F5, and F9 treatments.

### Histopathological Assessment and Immunohistochemical Analysis

**FIGURE 2:**
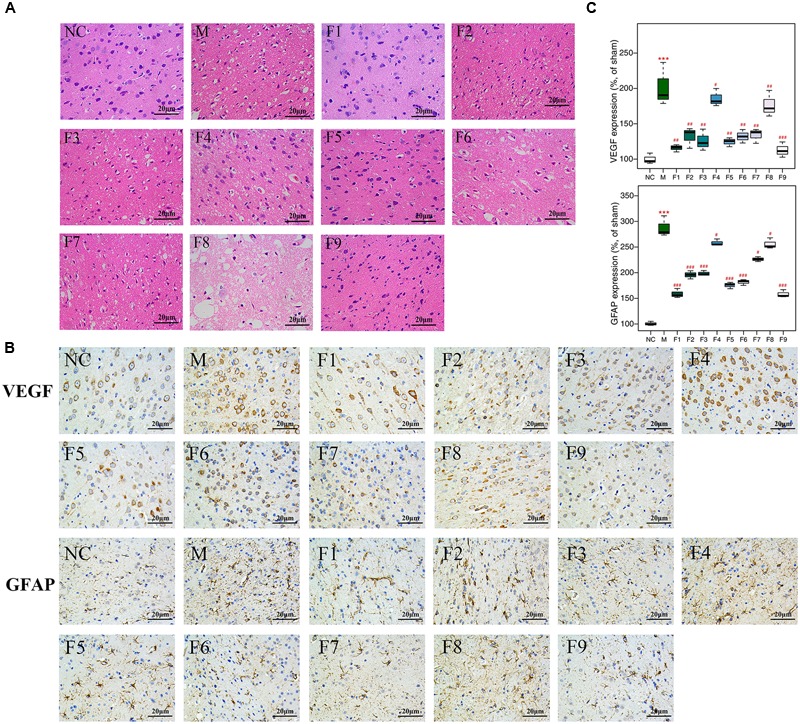
**H&E and immunohistochemical staining. (A)** Histopathological examination of brain tissues by H&E staining (×200, *n* = 4). **(B)** The expression of GFAP and VEGF in brain tissues of rats as determined by immunohistochemical staining (×400, *n* = 4). **(C)** The protein levels of GFAP and VEGF in brain tissues of rats determined by immunohistochemical staining. Results of quantitative analysis values are expressed as mean ±SD (*n* = 4). ^∗∗∗^*p* < 0.001 MCAO group vs. sham group; ^#^*p* < 0.05, ^##^*p* < 0.01, and ^###^*p* < 0.001 Treatment groups vs. MCAO group.

Apparent pathological changes occurred in cerebrum tissues of MCAO rats: destructed neuron structure, neuronal loss and numerous vacuolated spaces. F1, F5, and F9 treatments remarkably ameliorated pathological changes in the brain tissues of MCAO rats, while pathological abnormalities were occasionally observed for the F4 and F8 groups (**Figure 2A**).

The effects of F1–F9 on the expression of GFAP (the major protein constituent of glial intermediate filaments in differentiated fibrous and protoplasmic astrocytes of the central nervous system) (Xia et al., 2004) and VEGF (a key regulator of physiological and pathological angiogenesis) (Storkebaum et al., 2004) after I/R was investigated using immunohistochemical analysis. Compared with sham groups, the positive cells expression of GFAP and VEGF are significantly increased in MCAO rats. F1, F5, and F9 treatments notably reduced the levels of GFAP and VEGF activity in the ischemic stroke (**Figures 2B,C**).

### Method Validation

To determine the concentration of 23 endogenous AAs in rat brains quantitatively and simultaneously, a LC-QTOF-MS/MS analytical method was developed and validated. Supplementary Figure S10 displays the UV chromatogram, total ion chromatogram (TIC) and extract ion chromatogram (EIC) of 23 AAs standard solution. The peak of each AA was identified by the comparison of the molecular weights and retention times. The concentration range of all standard curves varied as shown in Supplementary Table S9. The correlation coefficient (*r*^2^) for all analyses was above 0.993, indicating a good linearity. The LLOQs of all AAs analyzed were about 1 μmol/L. The intra- and inter-day accuracy and precision of the method were summarized in Supplementary Table S10. The accuracy and precision of the present method strictly conformed to the criteria for the analysis of biological samples where the RSD determined at each level did not exceed 15%. The recoveries and matrix effects of the AAs during the sample preparation process were ranged from 86 to 108% (Supplementary Table S11), which showed that this method gave acceptable recovery and was with no obviously matrix effect. The results from all stability tests are presented in Supplementary Table S12, which demonstrated a good stability of AAs over all steps of the determination at 4°C. The samples were stable after three freeze-thaw cycles and freezing for 2 weeks at -80°C. The RSD of reproducibility did not exceed 15%; the method, therefore, was proved to be reliable and applicable to routine analysis.

### Identification of Significant Metabolites through Global Metabolomics

#### Multivariate Analysis of ^1^H NMR Data of All Groups

**FIGURE 3:**
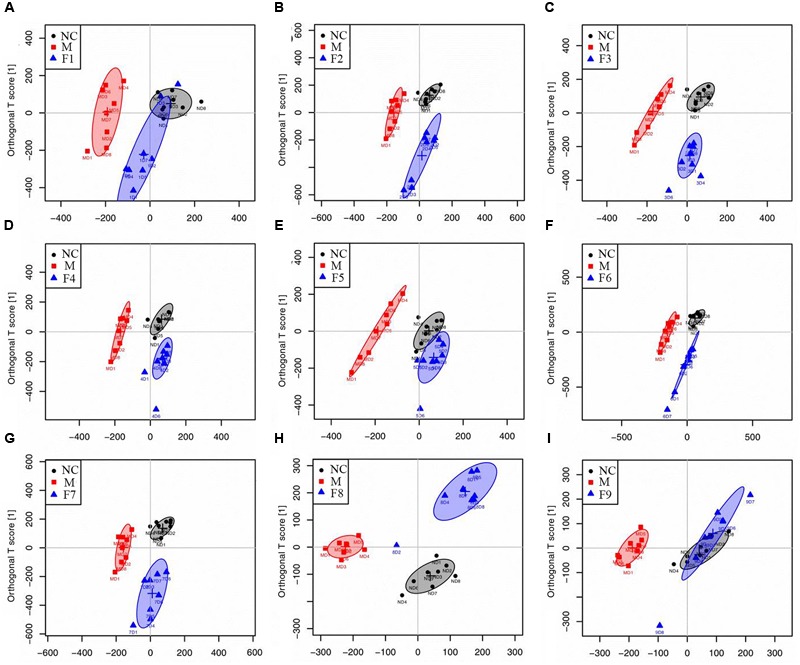
**Score plots for OPLS-DA analysis based on ^1^H NMR spectra.** Score plots for OPLS-DA analysis based on ^1^H NMR spectra of cerebrum extracts obtained from the sham, the MCAO and all treated rats (**A–I** for F1–F9, respectively).

^1^H NMR data of cerebrum extracts from the sham, MCAO and formulae 1–9 treated groups were analyzed by OPLS-DA model to investigate the effects of formulae 1–9 on MCAO rats. In the score plots, the showcased clusters corresponded to metabolic patterns in different groups with each point representing one sample. As shown in **Figure 3**, the global metabolic patterns of F1, F5, and F9 treated groups were clearly distinguishable with the MCAO group, and similar to the sham group. Though the groups treated with F2, F4, F6, F7, and F8 were distinguishable with the MCAO model group, they were also well separated from the control group, which reflected the bias of these therapies that radically affected the metabolic status of the control group. The fact that group NC and groups F1, F5, and F9 had a similar metabolic phenotype suggested that F1, F5, and F9 pretreatment prevented the changes of brain metabolites in MCAO rats with I/R injury and slightly affected the metabolic status of the control group. Above all, the F9 treatment group and the sham group were severely overlapped with each other, showcasing the great ability of F9 to protect against metabolic disturbance following the MCAO injury.

#### Metabolic Alterations in MCAO and Formulae 1–9 Treated Rats

To further investigate the metabolic perturbations induced by MCAO and find out metabolites that were directly associated with the treatment effects of formulae 1–9, ^1^H NMR data belonging to the MCAO group were compared with the sham group and F1–F9 groups by OPLS-DA analysis, individually.

**FIGURE 4:**
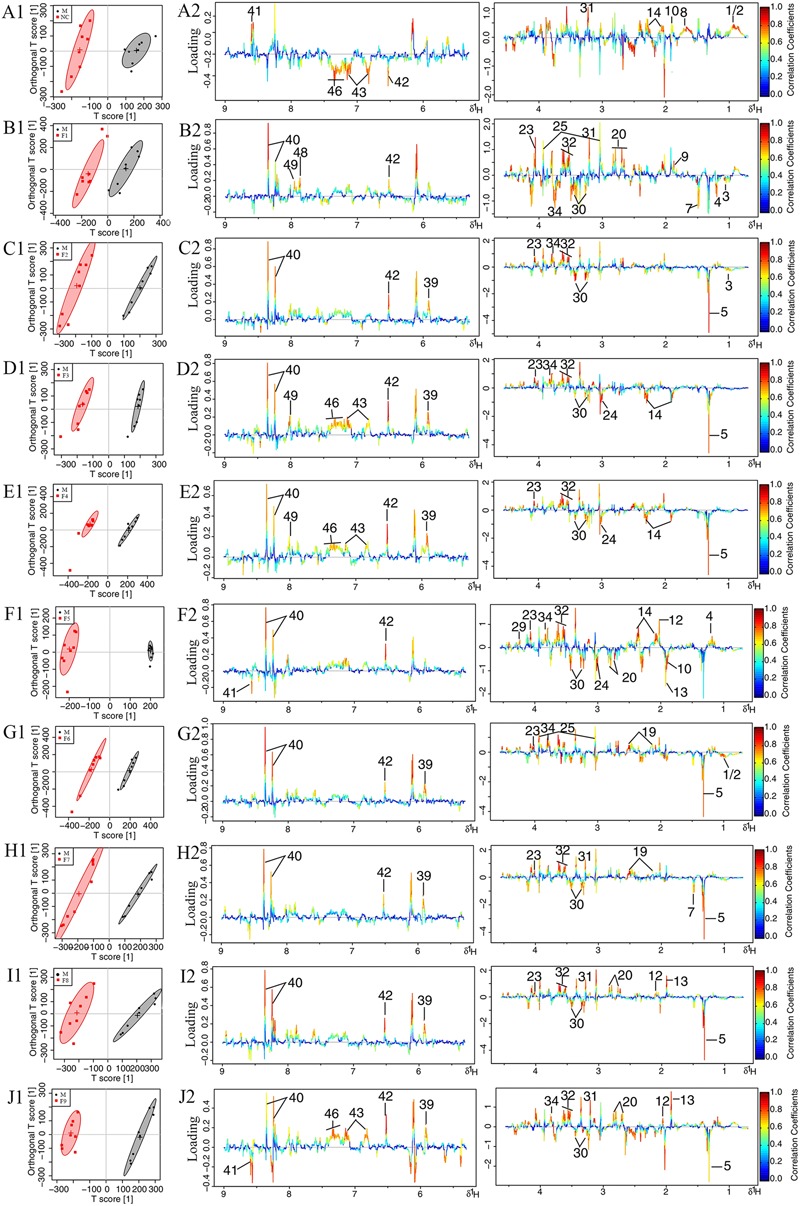
**Loading plots for OPLS-DA analysis based on ^1^H NMR spectra.** OPLS-DA analysis based on ^1^H NMR spectra of cerebrum extracts obtained from the sham, the MCAO and alltreated rats. Score plots **(A1–J1)** and the loading plots of OPLS-DA **(A2–J2)** were analyzed after the removal of H_2_O signals. Metabolite variation was visualized by the color-coded loading plots.

The score plots for cerebrums presented a clear clustering of the sham and MCAO groups with a well goodness of fit (*R*^2^*Y* = 0.91, *Q*^2^*Y* = 0.82, **Figure 4** and Supplementary Figure S11A), indicating excellent models. Model validity was verified using permutation tests where the predictive capability of a model using real class assignments was compared with a number of models calculated after random permutation of the class labels. The diagnostic model statistics AUROC (Supplementary Figures S12A1–J1) and misclassification rate (Supplementary Figures S12 A2–J2) were calculated based on a 2,000 times permutation test. An empirical *p*-value was calculated by determining the number of times the permutated data yielded a better result than the one using the original labels. The observed statistic *p*-values via permutation testing were all less than 0.05, thus suggesting statistically significant separation of classes and the validity of the OPLS-DA model. OPLS-DA loading plots (**Figure 4**) and S-plots (Supplementary Figure S11B) were generated to identify the main metabolites responsible for the differentiation in the score plots. The color-coded loading plots were color-encoded according to the absolute correlation coefficient of each variable to grouping, a hot-colored signal (red) indicated more significant contribution to class separation than a cold-colored one (blue), and presented in a covariance-based pseudo-spectrum (Hamabe et al., 2005). S-plots were another way to identify significantly altered metabolites, which should be located in the upper right or lower left quadrant and farther away from the origin.

The loading plots and S-plots showed obvious increases of leucine, isoleucine, lactate, alanine, lysine, glutamate, GABA, glycine, glycerol, serine, histamine, methionine and phenylalanine, and significant decreases of NAA, aspartate, GSH, ascorbic acid, taurine, AMP, arginine, pyruvate and citrate in MCAO group as compared with the sham group.

#### Univariate Analysis of ^1^H NMR Data of All Groups

**FIGURE 5:**
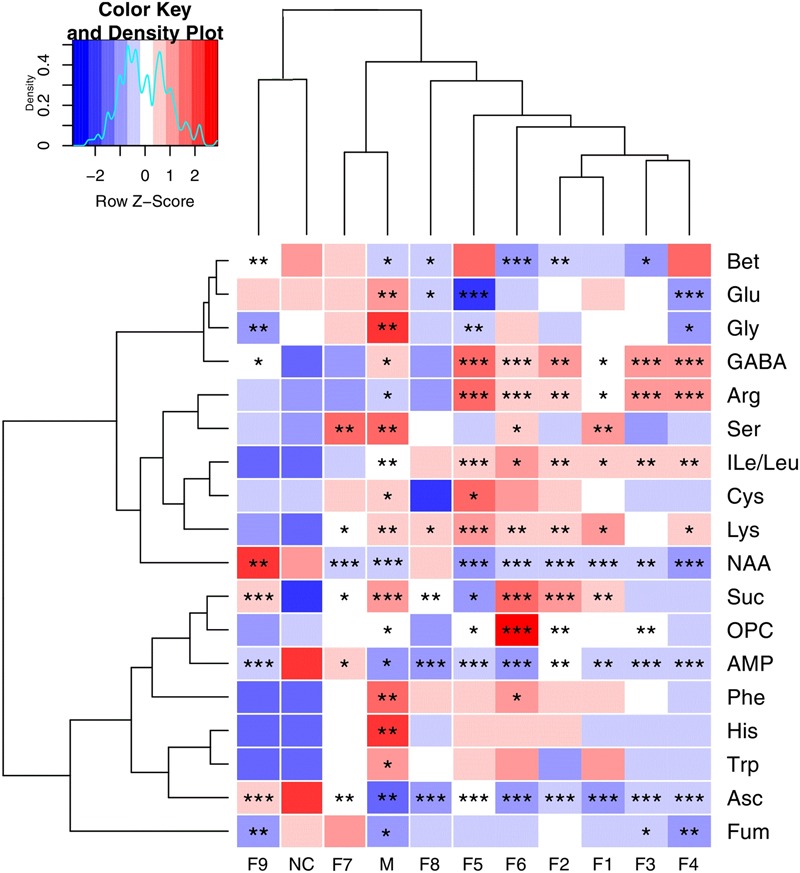
**Heatmap visualization of the metabolites in cerebral extracts.** Heatmap visualization of the *z*-scored levels of metabolites in cerebral extracts with stars denoting the significance. Rows represent metabolites and columns represent groups. Color key indicates metabolite quantities, white: no significant change, deep red: highest, deep blue: lowest. ^∗^*P* < 0.05, ^∗∗^*P* < 0.01, and ^∗∗∗^*P* < 0.001, all groups were compared with the sham group (NC).

These important differential metabolites identified by OPLS-DA loading plots and S-plots were further tested for their between-group difference using univariate analysis and visualized in the heatmap (**Figure 5**). There was no statistical difference between the F9 treatment group and sham group, which were clustered together, showcasing the great ability of F9 to protect against metabolic disturbance following the MCAO injury.

#### Metabolite Correlation Network Analysis in Global Metabolomics

**FIGURE 6:**
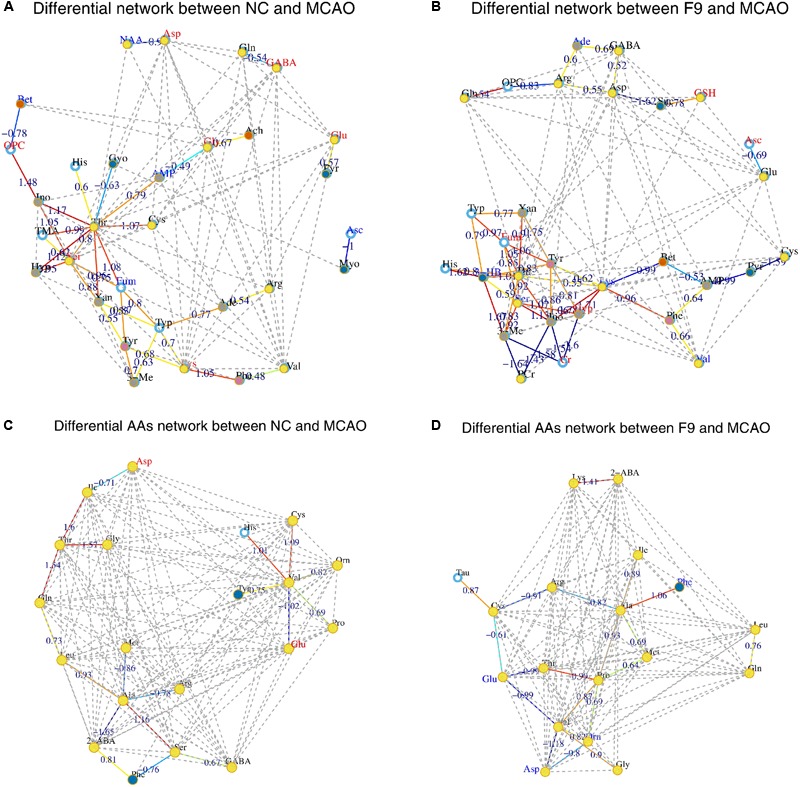
**Correlation networks of differential metabolites and amino acids (AAs) in cerebral extracts.** Differential network between NC and MCAO groups **(A)** and differential network between F9 and MCAO groups **(B)**; Differential network of AAs between NC and MCAO groups **(C)** and differential AAs network between F9 and MCAO groups **(D)**. Only correlations with absolute values of correlation coefficients greater than 0.3 and *p*-values < 0.05 were screened out. The nodes represent the metabolites, and the lines between the nodes indicate the biological relationships between the two corresponding metabolites. The red (blue) metabolites represent the upregulated metabolites (the downregulated metabolites) in MCAO rats compared with the sham rats or in drug-treated rats compared with the MCAO rats. The solid lines between the molecules indicate a correlation between the molecules, the line colors of red and blue display positive and negative relationships, respectively. Metabolites of similar structures were connected by the dotted lines, indicating a possible biochemical reaction between the molecules.

Instead of only investigating the individual metabolite variation, metabolic correlation networks were performed to decipher the biological correlation between the candidate biomarkers and illustrate the links in the potential biomarker metabolic pathway. The differential metabolic correlation networks were constructed based on the differential metabolites in sham and all treated groups (F1–9) compared to the MCAO group, and applied to further compare the outcome of sham and all treated groups (Supplementary Figure S13). Correlations between metabolites that exist in the networks are potentially more interesting and can offer insights into metabolic pathways that are altered between the sham and MCAO groups, the F1–F9 treatments and MCAO groups, respectively. For the differential network between NC and MCAO (**Figure 6A**), alteration of metabolic regulation is highlighted particularly well by threonine, which was located in the center of the network and highly correlated with many metabolites, including AMP, glycerol, histidine, inosine, TMA, serine, hypoxanthine, xanthine, fumarate, and cysteine. This suggested a considerable change in metabolic regulation occurred between the sham and MCAO groups. Some obvious differences were observed between the sham and F1–8 differential networks, however, the sham and F9 differential networks show several similarities. For the differential network between F9 and MCAO (**Figure 6B**), threonine, fumarate, tryptophan, lysine, serine, tyrosine were located in the center of the networks and highly correlated with many metabolites, including histidine, AMP, phenylalanine, inosine, creatine, and PCr. All the results suggested that AA metabolism accounted for a large proportion of the pathway alterations induced by I/R and might be responsible for the treatment effects of F9 in the ischemic stroke.

### Identification of Significant Metabolites through Targeted Metabolomics

#### Targeted Metabolomics Analyses of AAs in Cerebrum Tissues

**FIGURE 7:**
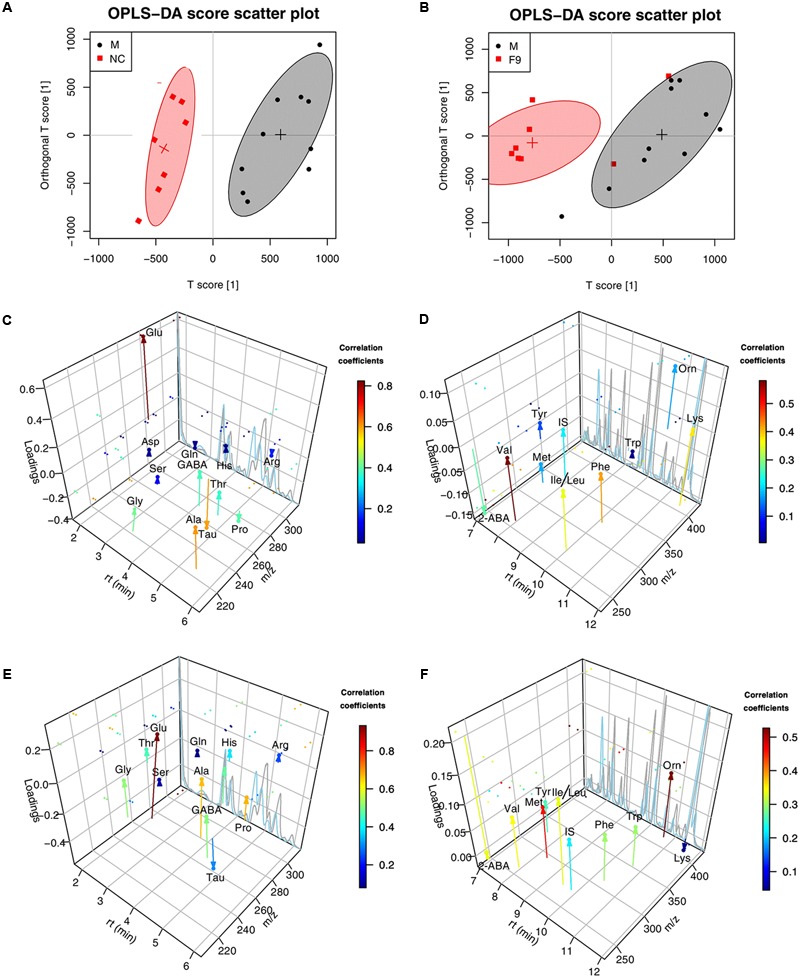
**The three-dimensional diagrams of OPLS-DA loading plots for AAs.** Score plots **(A,B)** and the three-dimensional diagrams of loading plots **(C–F)** for OPLS-DA analysis based on AAs targeted metabolomics analysis of cerebrum extracts obtained from the sham, the MCAO and all treated rats. **(C,D)** NC group VS. MCAO group; **(E,F)** F9-treated group VS. MCAO group.

The global metabolomics study showed that a few AAs play an important role in the metabolic correlation network. For the metabolic changes of AAs in the eleven groups of rats to be more comprehensively and accurately monitored, 23 AAs (Asp, Glu, Cys, Ser, Gln, Gly, His, Tau, GABA, Thr, Arg, Ala, Pro, 2-ABA, Tyr, Val, Met, Ile, Leu, Phe, Trp, Orn, Lys) were accurately quantified through a subsequent targeted metabolomics analysis. A supervised OPLS-DA model with the 23 AAs was successfully established to discriminate the sham and model groups. The OPLS-DA score plots (Supplementary Figure S14) of AAs showed a clear differentiation between these two groups at 24 h after I/R, suggesting that marked changes in the levels of AAs occurred during the ischemic injury. In addition to NC group, only F9 group was completely separated from the M group, others were partially overlapped with the M group, demonstrating the severe AAs disturbance induced by MCAO could be ameliorated after F9 treatment. According to the three-dimensional diagrams of OPLS-DA loading plots (**Figure 7**), the concentrations of 14 AAs (Glu, Asp, Ile, Leu, GABA, Gly, Thr, Ala, Lys, Phe, Met, Trp, Ser, and Orn) were increased significantly, but the 2-ABA, Arg, Tau, and Pro levels dropped dramatically in the brain tissues after MCAO, compared with those of sham rats. Formula 9 significantly attenuated the increase in Glu, GABA, Gly, Asp, Phe and Thr and the decrease in 2-ABA, Arg and Pro induced by ischemic injury at 24 h after reperfusion. Above all, F9 had significant effects on the AA levels in rat brain tissues following MCAO. Glu, Asp, Thr, Phe, GABA, Gly, Ser, and Arg were identified as the biomarkers that may play a role in the anti-cerebral ischemia effects of F9.

#### Correlation Network Analysis of Amino Acids in Targeted Metabolomics

The metabolic AAs profiling data was further analyzed by correlation network analysis to decipher the biological correlation between the candidate AA biomarkers. As shown in Supplementary Figure S15, the differential networks of AAs were constructed based on the differential AAs in brain tissues of sham and F1–9 groups, compared to MCAO rats. Some clear differences were observed between the sham and F1–F8 differential networks, while the F9 differential network was similar to sham differential network.

For the NC differential network of AAs (**Figure 6C**), Thr, Val, and Ala were in the center of the network, and highly correlated with many AAs. A strong positive correlation was observed between Val and His, Val and Cys, Thr with Gln, Thr with Gly, Thr with Ile, Ala with Ser, respectively. Strong negative correlations among Val and Glu, Ala and 2-ABA were also observed. These correlations were absent for F1–8 differential networks but were still found in F9 differential network (**Figure 6D**). An example of AAs correlated in both NC and F9 differential networks was that Thr, Val, and Ala were in the center of the network, and have strong correlations with other AAs. Our results demonstrated that the perturbed correlation network of AAs in the MCAO rats could be partially rectified by F9.

## Discussion

In this study, the global and AAs-based targeted metabolomics approach integrated with metabolic correlation networks has been established to evaluate the treatment effects of nine groups (F1–F9) of different combination of HLJDD component herbs according to an orthogonal experimental design on ischemic stroke and to explore the underlying mechanisms. Our results strongly demonstrated that HLJDD with optimal proportion 6(Rc): 4(Rs): 1(Cp): 3(Fg), namely F9, exhibited the strongest therapeutic effects in the MCAO models among all treated groups (F1–F9), which could be ascribed to its modulation on AA metabolism. F9 had good pharmacological effects on ischemic stroke was also demonstrated by various indexes such as neurobehavioral evaluations, cerebral infarct assessments, biochemical evaluations, histological inspections and immunohistochemistry observations. Compared to HLJDD (F1) and other HLJDD variants (F2–F8), F9 have an increased proportion of *Rhizoma coptidis* and *Radix scutellariae*, which might be responsible for its superior efficacy over other combinations. The results of the present study indicate that increase of either *Rhizoma coptidis* (F2, F7, and F8) or *Radix scutellariae* alone (F6) has no apparent augmentation of the therapeutic effects, but increases of both *Rhizoma coptidis* and *Radix scutellariae* together (F9) could obviously enhance the efficacy in treating ischemic stroke.

The global metabolomics results combined with metabolic correlation network analysis revealed that AAs metabolism was significantly perturbed in MCAO rats. For the differential network between NC and MCAO, threonine was located in the center of the network and highly correlated with many metabolites, including AMP, glycerol, histidine, inosine, TMA, serine, hypoxanthine, xanthine, fumarate and cysteine, thus it might have biochemical significance. Among them, fumarate, the key intermediate of the TCA cycle, was highly correlated with five metabolites (threonine, serine, tyrosine, and tryptophan), however, these correlations were absent for MCAO rats, indicating a shift of energy production from aerobic respiration toward anaerobic glycolysis in MCAO rats. AMP, inosine, hypoxanthine, and xanthine are associated with energy metabolism. ATP produced by energy-inefficient glycolysis could not suffice energy need of brain, which thus necessitated the catabolism of ATP to adenosine, and further to inosine, hypoxanthine and xanthine to provide more energy (Barsotti and Ipata, 2004).

Notably, strong negative correlations were observed among Cr, PCr, 3-Me, Ino, and Hyp in differential network between F9 and MCAO, which suggested that F9 greatly improved the damaged energy metabolism in MCAO rats. The Cr-PCr system, through the creatine kinase (CK) reaction, played crucial roles in maintaining a constant ATP level (Chen et al., 2012). With the improved energy supply in F9 group, the other energy production means were no longer necessary as exemplified by the elevated Cr level, and reduced ketone body (3-HB) level in brains of F9 rats, as compared with those in the MCAO rats.

Glutathione (a tripeptide consisting of glutamate, cysteine, and glycine) and ascorbate were also screened as key nodes in the sham compared MCAO network. They are low-molecular weight ROS scavengers and critical to attenuate the injury induced by I/R (Chamorro et al., 2002; Ozkul et al., 2007; Dasuri et al., 2013). It is well known that ischemic stroke is a disease in which free radicals are involved (Cherubini et al., 2005; Crack and Taylor, 2005). The lower level of ascorbate in MCAO rats implied an increased generation of free radicals. Compared with the MCAO rats, GSH and ascorbate were significantly increased in brains of F9 rats, which was helpful for the body to counteract ROS and ameliorate the status of oxidative stress induced by I/R injury. Consistent with these results, F9 significantly lowered the increased levels of oxidative stress markers (NO and MDA), and enhanced the activities of antioxidases (SOD, CAT, and GPx) and the level of GSH, as compared with the MCAO group.

Excitatory AAs (glutamate and aspartate) and inhibitory AAs (GABA, glycine) were proved to be key nodes in the differential network between sham and MCAO groups. The MCAO rats showed significant increase in both excitatory AAs (glutamate) and inhibitory AAs (GABA, glycine) levels in cerebrum, indicating cerebrum damage. Since the discovery of ischemia-evoked releases of glutamate in the rat hippocampus, evidence has accumulated during the past two decades showing that the excessive release of excitatory AAs such as glutamate is the pathological mechanism behind ischemic cerebrum damage (Saransaari and Oja, 1998; Nishizawa, 2001). Inhibitory AAs, such as GABA and glycine, have also been reported to be released during cerebral ischemia as a protection to alleviate the severity of ischemic injury and to counteract the toxicity of excitatory AAs (Bie et al., 2007). It is well established that during brain ischemia, glutamate plays an important role in mediating neuronal damage, and higher glutamate concentrations in the blood and CSF are associated with an increased neurological deterioration after stroke in humans and rats (Castillo et al., 1997). In animal models of stroke, glutamate scavenging has provided promise as a potentially useful therapeutic intervention. After F9 treatment, glutamate, aspartate, GABA and glycine were restored to the normal levels, indicating that F9 could not only restore the increased levels of excitatory neurotransmitters in MCAO, but also is good at restoring the level of inhibitory neurotransmitters in MCAO.

Interestingly, we observed significantly decreased levels of another key node *N*-acetylaspartic acid (NAA) in cerebrum of MCAO rats. NAA, the second most concentrated molecule in the cerebrum after glutamate, is used as an indicator of neuronal death in early stage. Previous studies have furnished strong evidence to support the view that NAA is an *in vivo* maker of neuronal density and its reduction is related to neuronal damage and loss in many cerebral disorders (Demougeot et al., 2001; Moffett et al., 2007).

In summary, the integrated analyses of the global metabolomics and correlation networks provided evidence of energy imbalance, oxidative stress and a disordered AA metabolism in ischemia reperfusion injury. Among all the parameters, AA metabolism accounted for a large proportion of the pathway alterations induced by I/R and might be responsible for the treatment effects of F9 in the ischemic stroke.

A total of 23 endogenous AAs were then simultaneously determined in rat brain tissues based on HPLC-QTOF-MS/MS method. OPLS-DA analysis of the AAs profiles suggested better therapeutic effects of F9 on the treatment of MCAO rats. We observed significant correlations of AAs in sham differential networks that appear to be separated into three clusters, such as threonine cluster, alanine cluster and valine cluster. More importantly, threonine and valine were the hubs of both the global metabolic correlation network and the AAs metabolic correlation network, which complemented each other. These data provided further support for the major impact of a disordered AA metabolism on the development of ischemic stroke.

It is undoubtedly a new trial to explore the optimal proportional of Rc, Rg, Cp, and Gf in treating cerebral ischemia by examining the AAs metabolic profiling. The results from AAs-based targeted metabolomics complemented with NMR global metabolomics suggested that altered AA levels may serve as diagnostic biomarkers for stroke. AA metabolic profiling sufficed to diagnostically denote the therapeutic effects of combinational drugs on ischemic stroke.

So far, we proposed optimal proportion of the four component herbs in HLJDD as a combination drug for the first time with the assistance of orthogonal design and global and AAs-based targeted metabolomics methods. All of these results indicated that in the treatment of acute ischemic stroke, we should pay more attention to the AA metabolism. These findings suggested an association between disorders of AA metabolism and ischemic stroke, and that the hypothesis was strongly supported by earlier reports on excitotoxic AAs and the risk of ischemic stroke (Castillo et al., 1997). This integrated metabolomics approach provides a new method to investigate and understand the molecular mechanism of TCM, and AA metabolism could be promising targets to treat ischemic stroke and predict the prognosis of stroke disease.

## Conclusion

In this study, global and AAs targeted metabolomics approach combined with correlation network analysis was firstly used to explore the optimal proportional of the four components in HLJDD for the treatment of ischemic stroke, which disclosed AAs metabolism as the major targets underlying the treatment of stroke by HLJDD variants. These findings demonstrated a strong association between disorders of AA metabolism and ischemic stroke. Our study showed the potential and applicable of this integrated metabolomics approach in deciphering the complex mechanisms of TCM formula on the treatment of complicated diseases, which provided new means to assess the treatment effects of herb combinations.

## Author Contributions

QZ, JW, MY, and LK conceived the experiments and helped to coordinate support and funding. QZ performed the research and drafted the manuscript. SL, PL, DX, and YL participated in the experiments. JW analyzed the data and edited the paper. All authors read and approved the final manuscript.

## Conflict of Interest Statement

The authors declare that the research was conducted in the absence of any commercial or financial relationships that could be construed as a potential conflict of interest.

## Footnotes

1 http://cran.r-project.org/

2 http://stat.ethz.ch/R-manual/R-devel/library/stats/html/p.adjust.html

3 http://mmcd.nmrfam.wisc.edu/

4 http://www.hmdb.ca/
