# Supplementary material for: Optimization of Huang-Lian-Jie-Du-Decoction for Ischemic Stroke Treatment and Mechanistic Study by Metabolomic Profiling and Network Analysis
Source: Front Pharmacol. 2017 Mar 28;8:165. doi: 10.3389/fphar.2017.00165 (PMC5368223; doi:10.3389/fphar.2017.00165)
Supplement: Supplementary file 1 [file Table_1.PDF]

Optimization of Huang-Lian-Jie-Du-Decoction for ischemic stroke treatment and mechanistic study by metabolomic profiling and network analysis

Qian Zhang[1], Jun-Song Wang[2 *], Shan-Ting Liao[1], Pei Li[1], Ding-Qiao Xu[1], Yan Lv[1], Ming-Hua Yang[1], Ling-Yi Kong[1 *]

[1] State Key Laboratory of Natural Medicines, Department of Natural Medicinal Chemistry, China Pharmaceutical University, 24 Tong Jia Xiang, Nanjing, 210009, P.R. China

[2] Center for Molecular Metabolism, Nanjing University of Science and Technology, 222 Xiao Ling Wei Street, Nanjing, 210094, P.R. China

[*] Correspondence:
Lingyi Kong
cpu_lykong@126.com

Junsong Wang
wang.junsong@gmail.com

Table S1 The weight ratios and yields of formulae 1-9.

groups	Rhizoma Coptidis(g)	Radix Scutellariae(g)	Cortex Phellodendri(g)	Fructus Gardeniae(g)	Yield (%)
F1	240	160	160	240	26.48
F2	370	123	123	184	28.94
F3	142	188	188	282	27.5
F4	114	152	76	458	28.0
F5	126	84	337	253	27.25
F6	133	356	178	133	37.12
F7	356	118	237	89	29.06
F8	322	53	107	322	29.44
F9	343	229	57	171	30.50

Table S2 Compounds detected in the extracts of formulae 1-9 obtained by HPLC-QTOF-MS.

Peak	t_R (min)	Experimental M^+	Calculated M^+	Error (ppm)	Molecular formula	Proposed compound
**1**	10.741	342.1704	342.1700	-1.26	$C_{20}H_{24}NO_4$	Phellodendrine
**2**	11.117	342.1701	342.1700	-0.24	$C_{20}H_{24}NO_4$	Magnoflorine
**3**	11.767	342.1699	342.1700	0.33	$C_{20}H_{24}NO_4$	Cyclanoline
**4**	15.652	322.1070	322.1074	1.05	$C_{19}H_{16}NO_4$	Berberrubine
**5**	17.398	338.1384	338.1387	0.88	$C_{20}H_{19}NO_4$	Jatrorrhizine / Columbamin
**6**	17.550	336.1232	336.1230	-0.48	$C_{20}H_{17}NO_4$	Epiberberine
**7**	17.812	320.0918	320.0917	-0.35	$C_{19}H_{14}NO_4$	Coptisine
**8**	18.664	350.1388	350.1387	-0.2	$C_{21}H_{20}NO_4$	Jatrorrhizine
**9**	19.335	352.1540	352.1543	0.84	$C_{21}H_{22}NO_4$	Palmatine isomers
**10**	19.618	352.1545	352.1543	-0.36	$C_{21}H_{22}NO_4$	Palmatine
**11**	20.050	336.1235	336.1230	-1.37	$C_{20}H_{18}NO_4$	Berberine

Table S3 Compounds detected in the extracts of HLJDD obtained by HPLC-QTOF-MS.

Peak	t_R (min)	Experimental $[M+H]^+$	Calculated $[M+H]^+$	Error (ppm)	Molecular formula	Proposed compound
**12**	12.416	369.1186	369.1180	-1.53	$C_{17}H_{20}O_9$	Chlorogenic acid

						methylester
**13**	15.246	368.1183	368.1180	-0.85	$C_{17}H_{20}O_9$	Methylchlorogenate
**14**	16.094	352.1183	352.1179	-1.04	$C_{20}H_{17}NO_5$	Berlambine
**15**	16.765	549.1607	549.1603	-0.81	$C_{26}H_{28}O_{13}$	Chrysin 6-C-arabinoside 8-C-glucoside
**16**	24.955	447.0925	447.0922	-0.69	$C_{21}H_{18}0_{11}$	Baicalin
**17**	29.555	447.0923	447.0922	-0.3	$C_{21}H_{18}O_{11}$	Baicalin isomers
**18**	31.186	461.1080	461.1078	-0.39	$C_{22}H_{20}O_{11}$	Oroxylin A 7-O-glucuronide
**19**	32.436	461.1082	461.1078	-0.77	$C_{22}H_{20}O_{11}$	Wogonoside
**20**	36.179	471.2012	471.2013	-0.16	$C_{26}H_{30}O_8$	Obaculactone
**21**	36.992	285.0755	285.0757	0.95	$C_{16}H_{12}O_5$	Wogonin
**22**	37.014	375.1082	375.1074	0.56	$C_{19}H_{18}O_8$	5,7-Dihydroxy-6,8-dimethoxyflavone
**23**	37.330	352.1182	352.1179	-0.78	$C_{20}H_{17}NO_5$	8-Oxyberberine
**24**	37.543	285.0755	285.0757	0.8	$C_{16}H_{12}$O5	Wogonin isomers
**25**	43.300	285.0766	285.0757	-2.83	$C_{16}H_{12}O_5$	Oroxylin A

Table S4 Compounds detected in the extracts of HLJDD obtained by HPLC-QTOF-MS.

Peak	t_R (min)	Experimental $[M+Na]^+$	Calculated $[M+Na]^+$	Error (ppm)	Molecular formula	Proposed compound
**26**	13.213	411.1259	411.1262	0.73	$C_{17}H_{24}O_{10}$	Jasminoidin

Table S5 Compounds detected in the extracts of HLJDD obtained by HPLC-QTOF-MS

Peak	t_R (min)	Experimental $[M-H]^-$	Calculated $[M-H]^-$	Error (ppm)	Molecular formula	Proposed compound
**27**	11.710	353.0875	353.0878	0.83	$C_{16}H_{18}O_9$	Chlorogenic acid
**12**	12.474	367.1028	367.1035	1.89	$C_{17}H_{20}O_9$	Chlorogenic acid methylester
**28**	15.295	367.1031	367.1035	0.87	$C_{17}H_{20}O_9$	3-O-Feruloylquinic acid
**29**	16.730	547.1455	549.1457	0.4	$C_{26}H_{28}O_{13}$	Puerarin 6"-O-xyloside
**30**	19.802	695.2186	695.2193	1.03	$C_{32}H_{40}O_{17}$	6”-*0*-trans-p-coumaroylge-nipin gentiobioside
**31**	21.726	551.2133	551.2134	0.21	$C_{27}H_{36}O_{12}$	Quercetin-3-o-rutinose

**16**	25.073	445.0774	445.0776	-0.9	$C_{21}H_{18}O^{11}$	Baicalin
**18**	31.703	429.0824	429.0827	0.83	$C_{21}H_{18}O_{10}$	Chrysin-7-o-glucoronide
**32**	32.074	475.0877	475.0882	1.11	$C_{22}H_{20}O_{12}$	5,6,7-trihydroxy-8-methoxyflavon-7-O-glucoronide
**33**	32.505	459.0930	459.0933	0.65	$C_{22}H_{20}O_{11}$	Wogonoside isomers
**34**	33.045	697.2707	697.2713	0.92	$C_{33}H_{46}O_{16}$	Luteolin-7-O-glucuronide
**35**	34.638	491.1187	491.1195	1.61	$C_{23}H_{24}O_{12}$	5,2,6’-trihydroxy-7,8-dimethoxyflavon-2’-O -D-glucopyranoside
**36**	37.012	283.0609	283. 0612	1.22	$C_{16}H_{12}O_5$	Oroxylin A isomers
**21**	37.105	373.0927	373.0929	0.4	$C_{19}H_{18}O_8$	5,7-dihydroxy-6,8-dimethoxyflavone
**23**	37.634	283.0606	283.0612	1.97	$C_{16}H_{12}O_5$	Wogonin isomers

Table S6 Compounds detected in the extracts of HLJDD obtained by HPLC-QTOF-MS

**Peak**	**t_R (min)**	**Experimental $[M+COOH]^-$**	**Calculated $[M+COOH]^-$**	**Error (ppm)**	**Molecular formula**	**Proposed compound**
**37**	11.121	595.1873	595.1880	1.05	C23H34O15	Genipin-1-β-D-gentiobioside
**26**	13.058	433.1350	433.1350	0.34	C17H24O10	Jasminoidin

Table S7 The orthogonal array $L_9(3^4)$ of Taguchi of HLJDD

groups	Rhizoma Coptidis	Radix Scutellariae	Cortex Phellodendri	Fructus Gardeniae
F1	1	1	1	1
F2	1	2	2	2
F3	1	3	3	3
F4	2	1	2	3
F5	2	2	3	1
F6	2	3	1	2
F7	3	1	3	2
F8	3	2	1	3
F9	3	3	2	1

Number 1 denotes remain the original ratios of the herbs in HLJDD formula unchanged;
Number 2 denotes half of the original ratios of the herbs in HLJDD formula;
Number 3 denotes double of the original ratios of the herbs in HLJDD formula.

Table S8 The assignment of metabolites in the cerebrum extracts of the sham, the MCAO and drugs-treated rats.

No.	Metabolite	Assignments	Chemical shift
1	Isoleucine	δCH_3, γCH_3, αCH	0.93(t), 1.0(d), 1.46(m)
2	Leucine	δCH_3, δCH_3, γCH, αCH	0.94(t), 0.96(t), 1.71(m), 3.74(m)
3	Valine	γCH_3, γCH_3	0.98(d), 1.03(d), 2.26(m), 3.60(d)
4	3-Hydroxybutyrate	γCH_3, βCH, αCH_2	1.20(d), 2.31(m), 2.41(m), 4.16(m)
5	Threonine	CH_3, αCH, βCH	1.34(d), 3.59(d), 4.26(m)
6	Lactate	CH_3, CH	1.32(d), 4.11(q)
7	Alanine	βCH_3, αCH	1.47(d), 3.78(q)
8	Lysine	δCH_2	1.48(m), 1.73(m), 1.91(m), 3.03(t), 3.76(t)
9	Arginine	γCH_2, βCH_2	1.78(m), 1.95(m)
10	GABA	αCH_2, βCH_2, γCH_2	1.91(m), 2.3(t), 3.02(t)
11	Acetate	CH_3	1.91(s)
12	NAA	CH_3, CH_2, CH	2.03(s), 2.51(m), 2.7(m), 4.4(m)
13	NAG	CH_3, CH_2, CH_2, CH	2.01(s), 2.32(m), 1.9-2.1(m), 4.2(m)

14	Methionine	S-CH_3, β-CH_2, S-CH_2, α-CH	2.14(s), 2.16(m), 2.65(t), 3.86(t)
15	Glutamate	βCH_2, βCH_2, γCH_2, αCH	2.10(m), 2.14(m), 2.36(m), 2.50(m), 3.77(t)
16	Glutathione	S-CH_2, N-CH, N-CH_2, CH_2	2.14(m), 2.55(m), 2.95(m), 3.77(m), 4.56(t)
17	Pyruvate	CH_3	2.34(s)
18	Succinate	CH_2	2.43(s)
19	Glutamine	βCH_2, γCH_2, αCH	2.46(m), 3.77(t)
20	Aspartate	βCH_2, α-CH	2.68(m), 2.82(m), 3.91(m)
21	Citrate	1/2CH_2, 1/2CH_2	2.67(d), 2.80(d)
22	TMA	CH_3	2.88(s)
23	Creatinine	N-CH_3, N-CH_2-CO	3.01(s), 4.05(s)
24	Creatine	CH_2, CH_3	3.04(s), 3.93(s)
25	Phosphocreatine	CH_2, CH_3	3.04(s), 3.93(s)
26	Ethanolamine	O-CH, NH_2-CH_2	3.22(t), 3.97(t)
27	Choline	$N(CH_3)_3$, N-CH_2	3.25(s), 3.51(m)
28	Acetylcholine	N(CH3)$_3$, N-CH_2, O-CH_2, CH_3	3.21(s), 3.67(t), 4.51(t), 2.1(s)
29	O-phosphocholine	$N(CH_3)_3$, N-CH_2, O-CH_2	3.21(s), 3.57(t), 4.16(t)
30	Taurine	NH_2-CH_2, SO_3-CH_2	3.25(t), 3.43(t)
31	Betaine	$N(CH_3)_3$, CH_2	3.27(s), 3.90(s)
32	Myo-inositol	CH	3.27 (t), 3.53(dd), 3.62 (t), 4.05 (t)
33	Glycine	CH_2	3.57(s)
34	Glycerol	CH_2, CH	3.6(m), 3.8(m)
35	Ascorbate	CH_2, CH	3.74(d), 3.76(d), 4.03(m), 4.52(d)
36	Serine	CH_2, CH	3.85(m), 3.98(m)
37	Inosine	O-CH-N, N-CH=N, N-CH=N	6.10 (d), 8.23 (s), 8.34 (s)
38	Uracil	CH=CH-N	7.54(d), 5.79(d)
39	Uridine	H5, H6, H1'	5.8(d), 5.82(d), 7.81(d)
40	Adenosine	CH, CH	8.25 (s), 8.34 (s)
41	AMP	N=CH-N, N=CH-N	8.23 (s), 8.56 (s)
42	Fumarate	CH=CH	6.53(s)
43	Tyrosine	H3/H5, C5H/C6H	3.06(m), 3.20(m), 3.94(m), 6.91(d), 7.20(d)
44	Histamine	NH_2-CH_2,CH=C-CH_2, CH=N-CH	3.00(t), 3.29(t), 7.12(s), 7.93(s)
45	Tryptophan	CH=CH	3.49(m), 4.06(m), 7.21(t), 7.29(t), 7.33(s), 7.55(d), 7.74(d)
46	Phenylalanine	CH=CH	3.13(m), 3.28(m), 4.00(m), 7.33(m), 7.38(m), 7.43(m)

47	Nicotinurate	NH_2-CH, H2/H4/H5/H6	4.43(s), 8.07(m), 8.83(m), 9.11(s)
48	Xanthine	NH=CH-N	7.95(s)
49	3-Methylxanthine	NH=CH-N, N-CH_3	3.52(s), 8.02(s)
50	Hypoxanthine	NH=CH-N, N=CH-NH	8.18(s), 8.20(s)

Multiplicity: singlet (s), doublet (d), triplet (t), doublet of doublets (dd), quartets (q), multiplet (m).

Table S9 Calibration curve, correlation coefficient (r^2), test range, weight coefficient and LLOQ for 23 amino acids (n=6)

Amino acid	Calibration equations [a]	r^2	weight coefficient	Linear range (μmol/L)	LLOQ [b] (μmol/L)
Asp	Y＝0.3757X-0.0615	0.9982	1/x	15.625-2000	2
Glu	Y＝0.0867X-0.3566	0.0106	1/x	15.625-2000	2
Cys	Y＝0.3678X-0.0623	0.9955	1/x	15.625-2000	2
Ser	Y＝5.5294X+0.8267	0.9962	1/x	15.625-2000	2
Gln	Y＝0.8786X-0.1338	0.9979	1/x	15.625-2000	2
Gly	Y＝0.2973X-0.0606	0.9981	1/x	15.625-2000	2
His	Y＝5.7473X-0.6914	0.9935	1/x	15.625-2000	2
Tau	Y＝1.1480X-0.2664	0.9962	1/x	15.625-2000	20
GABA	Y＝0.7498X-0.1570	0.9964	1/x	156.25-5000	2
Arg	Y＝2.7194X-0.4355	0.9948	1/x	15.625-2000	2
Thr	Y＝0.3970X-0.0715	0.9938	1/x	15.625-2000	2
Ala	Y＝0.3899X-0.0719	0.9961	1/x	15.625-2000	2
Pro	Y＝2.0431X-0.4189	0.9988	1/x	15.625-2000	2
2-ABA	Y＝1.4001X-0.3629	0.9972	1/x	15.625-2000	2
Tyr	Y＝2.9025X-0.7862	0.9938	1/x	15.625-2000	2
Val	Y＝0.8716X-0.9930	0.9943	1/x	15.625-2000	2
Met	Y＝2.2768X-0.3594	0.999	1/x	15.625-2000	2
Ile	Y＝4.4753X-0.5535	0.9984	1/x	15.625-2000	2
Leu	Y＝5.2479X-0.9122	0.9953	1/x	15.625-2000	2
Phe	Y＝0.4165X-0.1111	0.9962	1/x	15.625-2000	2
Trp	Y＝0.0703X-0.0070	0.9941	1/x	15.625-2000	2
Orn	Y＝0.4539X-0.0220	0.997	1/x	15.625-2000	2
Lys	Y＝0.3917X-0.0830	0.9964	1/x	15.625-2000	2

[a] y is the peak area in LC-QTOF-MSMS for 23 amino acids respectively; x is the compound amount injected

[b] LLOQ refers to the lower limits of quantitation.

Table S10 Precision and accuracy of 23 amino acids in rat brain tissues (n=6)

Amino acid	Concentration (mmol/L)	Precision RSD (%)		Accuracy RE (%)	
		Intra-day precision	Intra-day precision	Intra-day precision	Intra-day precision
Asp	0.0390625	8.66	8.02	6.46	7.96
	0.3125	8.17	8.52	6.55	8.06
	1.25	7.49	7.67	6.73	3.52
Glu	0.0390625	9.76	8.76	10.09	11.78
	0.3125	8.01	8.13	7.95	9.49
	1.25	7.45	7	-6.22	7.72
Cys	0.0390625	7.56	7.09	6.42	7.92
	0.3125	9.64	7.86	-9.03	-4.67
	1.25	10.73	3.46	5.95	6.77
Ser	0.0390625	7.71	7.77	7.28	8.18
	0.3125	8.39	9.09	9.23	10.92
	1.25	6.86	7.79	-6.55	-8.06
Gln	0.0390625	5.23	-5.21	4.43	2.78
	0.3125	6.42	6.39	6.42	7.57
	1.25	4.13	7.2	3.26	-5.06
Gly	0.0390625	7.45	7.78	7.05	8.59
	0.3125	6.46	7.36	5.77	7.22
	1.25	7.74	7.79	7.35	8.86
His	0.0390625	7.35	8.37	-2.96	-1.92
	0.3125	7.24	8.8	7.95	9.53
	1.25	7.45	8.68	8.85	9.59
Tau	0.0390625	11.82	10.62	13.25	5.71
	0.3125	10.85	2.66	4.55	5.99
	1.25	8.049	5.07	4.01	6.118
GABA	0. 390625	6.31	5.85	-4.28	-3.08
	3.125	10.26	8.84	10.02	12.32
	12.5	7.34	6.69	5.84	7.28
Arg	0.0390625	9.65	9.2	10.95	12.17
	0.3125	7.83	8.18	7.75	9.37
	1.25	7.23	6.99	5.83	-7.35
Thr	0.0390625	7.78	8.3	7.45	9.47
	0.3125	6.16	7.79	5.97	7.42
	1.25	5.91	6.97	-4.87	-3.97
Ala	0.0390625	7.98	7.8	7.53	9.09
	0.3125	7.57	8.64	8.05	9.65
	1.25	6.94	7.58	6.4	7.29
Pro	0.0390625	7.46	8.55	7.88	-9.46
	0.3125	7.04	8.26	7.21	8.74
	1.25	7.68	9.26	8.86	10.44

	0.0390625	7.6	7.56	6.94	8.48
2-ABA	0.3125	8.75	8.55	8.97	10.62
	1.25	8.05	7.94	7.75	9.31
	0.0390625	6.68	8.57	7.25	8.77
Tyr	0.3125	7.57	8.04	7.45	8.99
	1.25	6.11	8.14	6.25	7.77
	0.0390625	8.63	8.53	8.85	10.49
Val	0.3125	6.24	7.57	5.75	7.26
	1.25	8.67	7.96	8.33	9.49
	0.0390625	8.05	6.16	5.85	7.33
Met	0.3125	7.26	5.44	-4.45	5.81
	1.25	7.41	8.44	7.74	9.28
	0.0390625	6.08	7.26	5.32	6.76
Ile	0.3125	8.2	9.27	9.22	10.92
	1.25	8.14	7.5	7.33	8.49
	0.0390625	6.45	3.91	-2.59	-1.57
Leu	0.3125	8.78	4.83	-0.57	0.55
	1.25	9.62	5.72	0.14	1.31
	0.0390625	7.21	8.06	7.14	8.68
Phe	0.3125	8.29	8.85	8.91	10.54
	1.25	7.39	8.54	7.79	9.37
	0.0390625	5.35	2.57	-0.25	0.89
Trp	0.3125	4.36	7.61	-4.39	-3.47
	1.25	3.38	4.84	-5.24	-4.36
	0.0390625	8.3	7.93	7.15	9.53
Orn	0.3125	7.12	7.43	6.43	7.79
	1.25	7.48	8.38	7.74	9.28
	0.0390625	6.22	7.26	5.45	6.89
Lys	0.3125	7.95	7.24	6.96	8.44
	1.25	6.16	8.25	6.34	7.88

Table S11 Recovery and matrix effect for 23 amino acids in rat brain tissues (n=6)

Amino acid	Nominal concentration (mmol/L)	Recovery (Mean ± SD, %)	RSD (%)	Matrix effect (Mean ± SD, %)	RSD (%)
	0.0390625	96.52±4.82	9.26	87.92±4.82	4.73
Asp	0.3125	97.12±3.29	8.87	90.21±4.56	4.49
	1.25	96.90±6.96	8.29	98.90±5.73	4.95
	0.0390625	100.20±2.43	10.36	96.45±3.45	5.38
Glu	0.3125	97.87±4.16	8.81	89.56±5.45	4.45
	1.25	98.71±5.46	4.75	98.67±5.23	8.15
Cys	0.0390625	94.27±4.96	4.18	109.70±7.73	8.36

	0.3125	102.70±5.20	5.32	100.78±5.36	10.64
	1.25	95.97±6.24	5.09	101.78±4.37	11.83
	0.0390625	95.98±4.20	4.25	98.56±4.35	8.41
Ser	0.3125	96.64±6.18	9.29	90.56±4.56	4.61
	1.25	89.67±4.35	7.56	99.45±6.34	3.73
	0.0390625	98.07±5.96	8.15	98.26±4.72	4.05
Gln	0.3125	95.28±4.50	3.553	96.56±3.79	7.16
	1.25	97.12±5.46	4.257	100.46±8.92	8.4
	0.0390625	98.27±4.95	4.25	89.34±7.34	8.05
Gly	0.3125	97.26±5.28	3.82	102.34±6.24	7.94
	1.25	95.87±6.91	8.95	104.45±5.36	4.75
	0.0390625	95.66±3.23	13.02	101.16±5.47	6.01
His	0.3125	101.15±5.56	11.35	94.68±6.41	5.95
	1.25	98.16±2.51	4.65	96.81±5.67	8.89
	0.0390625	99.94±5.79	4.95	93.13±4.32	6.41
Tau	0.3125	101.91±6.40	6.93	97.34±4.56	11.86
	1.25	95.74±2.79	5.57	95.24±6.36	8.74
	0. 390625	101.37±7.28	6.85	86.34±4.57	10.15
GABA	3.125	96.85±5.89	5.85	82.45±6.34	8.63
	12.5	99.26±3.95	5.45	83.46±5.67	4.29
	0.0390625	97.65±6.17	9.43	98.67±7.89	3.38
Arg	0.3125	102.25±5.80	10.08	90.67±4.34	3.25
	1.25	95.37±6.54	8.26	101.56±7.46	4.39
	0.0390625	96.38±5.90	7.51	98.45±5.57	8.78
Thr	0.3125	98.12±4.84	5.65	89.56±5.63	8.37
	1.25	101.56±4.27	5.37	98.56±5.05	7.64
	0.0390625	102.45±4.57	5.53	94.45±4.65	8.26
Ala	0.3125	98.56±6.47	8.94	96.28±6.74	3.82
	1.25	89.56±7.75	9.98	101.65±3.26	4.24
	0.0390625	98.34±4.16	9.86	102.47±5.02	4.18
Pro	0.3125	86.71±4.28	11.15	100.16±5.53	4.81
	1.25	86.93±4.37	10.05	92.07±6.77	4.42
	0.0390625	98.29±4.16	5.24	99.28±4.74	7.38
2-ABA	0.3125	88.59±5.25	5.85	100.34±6.16	8.37
	1.25	87.63±4.81	4.85	100.67±3.20	6.71
	0.0390625	97.16±3.25	6.15	96.23±4.31	9.93
Tyr	0.3125	88.92±4.67	4.82	98.76±6.12	6.84
	1.25	96.34±4.02	10.87	101.17±5.85	4.75
	0.0390625	97.45±3.34	10.05	100.67±3.80	4.45
Val	0.3125	98.87±4.18	9.46	98.45±5.34	3.93
	1.25	89.67±4.45	9.61	101.15±5.39	4.05
	0.0390625	98.43±5.54	6.68	101.25±4.53	3.34
Met	0.3125	89.76±5.26	4.51	97.97±4.67	9.02
	1.25	98.59±3.45	4.77	96.76±3.95	8.54

	0.0390625	87.92±4.82	3.55	98.45±3.45	7.05
Ile	0.3125	97.83±4.83	9.58	96.40±5.92	4.89
	1.25	101.87±2.55	10.82	99.03±5.67	5.21
	0.0390625	97.38±4.71	7.91	98.66±3.45	3.95
Leu	0.3125	92.93±3.29	9.19	101.42±5.83	4.55
	1.25	95.26±6.78	8.19	96.35±5.33	4.05
	0.0390625	98.74±4.86	5.85	94.92±5.67	12.95
Phe	0.3125	88.12±3.57	8.18	96.23±3.31	2.38
	1.25	86.86±2.89	6.01	98.56±3.56	13.78
	0.0390625	98.50±4.72	6.27	89.45±5.34	9.13
Trp	0.3125	96.84±3.42	5.05	85.47±3.57	7.82
	1.25	97.27±5.48	11.08	99.46±3.56	8.28
	0.0390625	100.46±4.92	3.41	87.66±3.45	6.82
Orn	0.3125	97.95±3.32	4.35	89.34±5.24	8.75
	1.25	98.85±3.96	3.38	94.24±5.37	6.76
	0.0390625	101.57±5.55	9.26	89.35±5.36	4.73
Lys	0.3125	98.56±3.92	8.87	85.56±6.47	4.49
	1.25	96.86±4.29	8.29	87.67±3.35	4.95

Table S12 Stability of 23 amino acids in rat brain tissues under various storage conditions (n=6)

Amino acid	Spiked concentration (mmol/L)	Short-term stability		Long-term stability		Freeze-thaw stability		Stability Post-preparative stability	
		(RSD, %)	(RE, %)	(RSD, %)	(RE, %)	(RSD, %)	(RE, %)	(RSD, %)	(RE, %)
	0.0390625	7.06	6.86	8.83	5.92	8.64	-6.84	7.18	9.54
Asp	0.3125	6.67	6.93	8.15	6.06	9.14	6.93	7.28	9.69
	1.25	6.09	7.13	10.42	6.16	8.29	7.32	6.83	9.78
	0.0390625	7.96	10.69	8.67	9.28	9.38	10.18	7.74	13.57
Glu	0.3125	6.41	8.47	8.11	7.14	8.75	8.16	7.57	10.35
	1.25	6.05	6.59	8.22	5.74	7.62	6.63	6.23	8.88
	0.0390625	6.16	6.8	10.3	5.94	7.71	6.81	6.31	9.18
Cys	0.3125	7.84	9.57	11.39	8.36	8.48	9.17	6.99	12.25
	1.25	8.63	6.37	8.37	5.44	4.08	-5.82	3.94	7.75
	0.0390625	6.21	7.71	9.05	6.66	8.39	7.48	6.91	9.47
Ser	0.3125	6.79	9.78	7.52	8.46	9.71	9.12	8.01	12.58
	1.25	5.56	6.93	8.11	6.06	8.41	6.96	6.91	9.29
	0.0390625	6.05	7.43	7.12	6.46	8.4	7.34	6.92	9.85
Gln	0.3125	5.26	6.11	8.4	-5.3	7.98	6.22	6.55	8.33
	1.25	6.26	7.71	8.01	6.72	8.41	7.96	6.91	10.89
Gly	0.0390625	5.95	-3.13	7.9	-2.72	8.99	-1.62	7.43	-2.28

	0.3125	5.84	8.47	8.11	7.31	9.42	8.18	7.82	10.95
	1.25	6.03	9.81	12.48	8.12	9.3	8.24	7.72	11.02
	0.0390625	9.57	14.45	11.51	12.19	11.24	13.06	9.45	8.05
His	0.3125	8.78	4.23	8.70	-4.16	3.28	5.14	2.36	6.85
	1.25	6.51	4.06	6.97	3.68	5.69	5.26	4.53	7.07
	0.0390625	5.11	-4.55	10.92	-3.93	6.47	-2.64	5.25	-3.52
Tau	0.3125	8.31	10.62	8.37	9.24	9.46	10.59	7.86	14.18
	1.25	5.94	6.19	10.31	5.37	7.31	6.26	5.41	8.32
	0. 390625	7.81	11.67	8.49	10.04	9.82	10.46	8.18	13.95
GABA	3.125	6.34	8.25	7.89	7.13	8.8	8.05	7.02	10.55
	12.5	5.85	6.18	8.44	-5.36	7.61	6.31	6.22	8.25
	0.0390625	6.30	7.87	6.82	6.84	8.92	8.14	7.38	10.85
Thr	0.3125	4.98	6.32	2.57	-5.49	8.41	6.38	7.93	8.53
	1.25	4.78	-5.16	8.64	-4.48	7.59	-3.41	6.26	-4.55
	0.0390625	6.46	7.98	8.23	6.92	8.42	7.81	6.92	10.45
Arg	0.3125	6.13	8.53	7.60	7.46	9.26	8.29	7.66	11.09
	1.25	5.62	6.84	8.12	5.88	8.2	6.24	3.72	8.38
	0.0390625	6.04	8.35	7.70	7.24	9.17	8.13	7.65	10.79
Ala	0.3125	5.74	7.64	8.34	6.63	8.88	7.51	7.35	10.05
	1.25	6.28	9.36	8.26	8.15	9.88	-8.97	8.21	12.06
	0.0390625	6.15	7.34	9.41	6.38	8.18	7.29	6.72	9.72
Pro	0.3125	7.05	9.52	8.71	8.25	9.17	9.13	7.67	12.23
	1.25	6.52	8.15	7.34	7.13	8.56	-3.58	3.06	4.75
	0.0390625	5.41	7.65	8.23	6.67	9.19	7.54	7.62	10.05
2-ABA	0.3125	6.13	7.97	6.77	6.84	8.66	7.73	5.15	10.33
	1.25	4.94	6.65	9.29	5.75	8.76	6.68	7.24	-8.95
	0.0390625	6.93	9.31	6.90	8.12	9.15	9.02	1.59	12.35
Tyr	0.3125	5.04	6.05	9.33	5.29	8.19	6.24	6.73	8.39
	1.25	7.07	8.89	8.71	7.66	8.58	8.16	7.04	10.95
	0.0390625	6.55	6.21	7.92	-5.32	6.78	6.30	5.48	8.45
Val	0.3125	5.86	4.71	8.07	-4.94	6.06	-4.99	4.84	6.65
	1.25	6.01	8.24	6.74	7.18	9.06	7.98	7.51	10.72
	0.0390625	4.92	5.63	8.86	-4.89	7.88	-5.81	6.46	7.74
Met	0.3125	6.64	9.77	8.80	8.48	9.89	9.39	8.25	12.58
	1.25	6.59	7.76	7.11	6.74	8.12	7.30	6.67	9.75
	0.0390625	5.22	-2.74	9.44	2.38	4.53	-1.35	3.47	-1.85
Ile	0.3125	7.11	-6.04	10.28	1.52	5.45	3.473	4.29	4.63
	1.25	7.72	2.14	7.87	8.12	6.34	1.12	5.09	-1.5
	0.0390625	5.81	7.84	8.95	-6.56	8.68	7.46	7.17	9.98
Leu	0.3125	6.92	9.46	8.05	8.19	9.47	9.06	3.87	12.12
	1.25	5.99	8.74	6.01	7.16	9.16	8.05	7.64	10.77
	0.0390625	4.35	-7.65	5.02	-0.23	3.19	4.76	2.28	-1.02
Phe	0.3125	3.16	-4.64	4.04	-4.03	8.23	-2.98	6.77	-3.99
	1.25	2.78	-5.55	8.96	-4.88	5.46	-3.74	4.3	-5.04

	0.0390625	6.74	7.79	7.78	6.58	8.55	8.19	7.05	10.95
Trp	0.3125	5.72	6.81	8.14	5.91	8.05	6.69	6.61	8.95
	1.25	6.08	8.2	6.88	7.18	9.34	7.98	7.45	10.62
	0.0390625	5.02	5.77	8.61	5.04	7.88	5.92	6.46	7.92
Orn	0.3125	6.45	7.37	6.82	6.43	7.86	7.25	6.44	9.76
	1.25	4.96	6.72	8.58	5.83	8.87	6.77	7.34	9.06
	0.0390625	6.35	6.89	10.36	6.86	6.54	5.84	5.27	-3.66
Lys	0.3125	7.86	8.46	9.72	8.42	7.87	8.46	6.45	9.97
	1.25	7.34	5.44	7.66	9.48	7.39	8.24	6.03	1.35
