# Supplementary material for: Optimization of Huang-Lian-Jie-Du-Decoction for Ischemic Stroke Treatment and Mechanistic Study by Metabolomic Profiling and Network Analysis
Source: Front Pharmacol. 2017 Mar 28;8:165. doi: 10.3389/fphar.2017.00165 (PMC5368223; doi:10.3389/fphar.2017.00165)
Supplement: Supplementary file 2 [file Image_1.PDF]

Optimization of Huang-Lian-Jie-Du-Decoction for ischemic stroke treatment and mechanistic study by metabolomic profiling and network analysis

Qian Zhang[1], Jun-Song Wang[2 *], Shan-Ting Liao[1], Pei Li[1], Ding-Qiao Xu[1], Yan Lv[1], Ming-Hua Yang[1], Ling-Yi Kong[1 *]

[1] State Key Laboratory of Natural Medicines, Department of Natural Medicinal Chemistry, China Pharmaceutical University, 24 Tong Jia Xiang, Nanjing, 210009, P.R. China

[2] Center for Molecular Metabolism, Nanjing University of Science and Technology, 222 Xiao Ling Wei Street, Nanjing, 210094, P.R. China

[*] Correspondence:
Lingyi Kong
cpu_lykong@126.com

Junsong Wang
wang.junsong@gmail.com

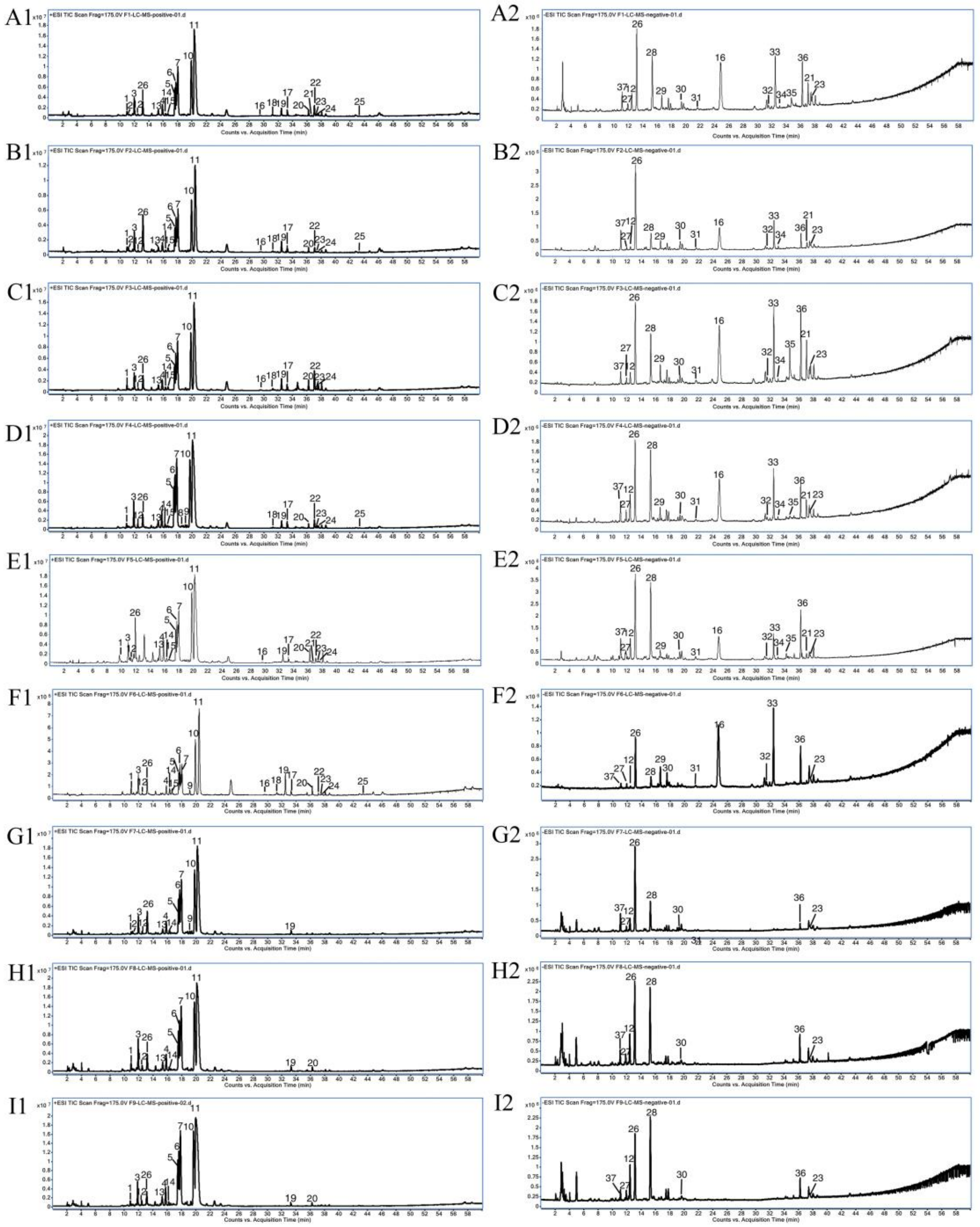

Figure S1 Profile of TIC chromatograms of formulae 1-9 extracts

Total ion current (TIC) chromatograms of F1-F9 extracts analyzed by HPLC-QTOF-MS in a positive ion mode (**A1-I1** for F1-F9, respectively) and a negative ion mode (**A2-I2** for F1-F9, respectively). Peaks 1-26 listed in Tables S2-4. Peaks 27-37 listed in Tables S5-6. The HPLC-QTOF-MS analysis was made using an Agilent 1290 infinity LC system connected to a 6520 quadrupole time-of-flight mass spectrometer. Chromatographic separation was performed on Shimadzu VP-ODS column (250×4.6 mm I.D., 5 μm particle size, Shimazdu, Kyoto, Japan) with a solvent flow rate of 1 ml/min at a temperature of 30 ℃. The mobile phase was composed of 10 mmol/L ammonium acetate titrated with acetic acid to pH 3.0 (A) and acetonitrile (B). The solvent gradient adopted was as follows: 0-4 min, 10% B; 4-15 min, 10-26%

B; 15-27 min, 26-28% B; 27-35 min, 28-70% B; 35-55 min, 70-90% B; 55-60 min, 90% B. The positive and negative ion ESI-MS experiments were conducted using conditions as follows: drying gas temperature, 325 ℃; drying gas (N^2) flow rate, 10 L/min; nebulizer, 45 psi; capillary voltage, 4000 V (+) and 3500 V (-); skimmer, 65 V; fragmentor, 175 V.

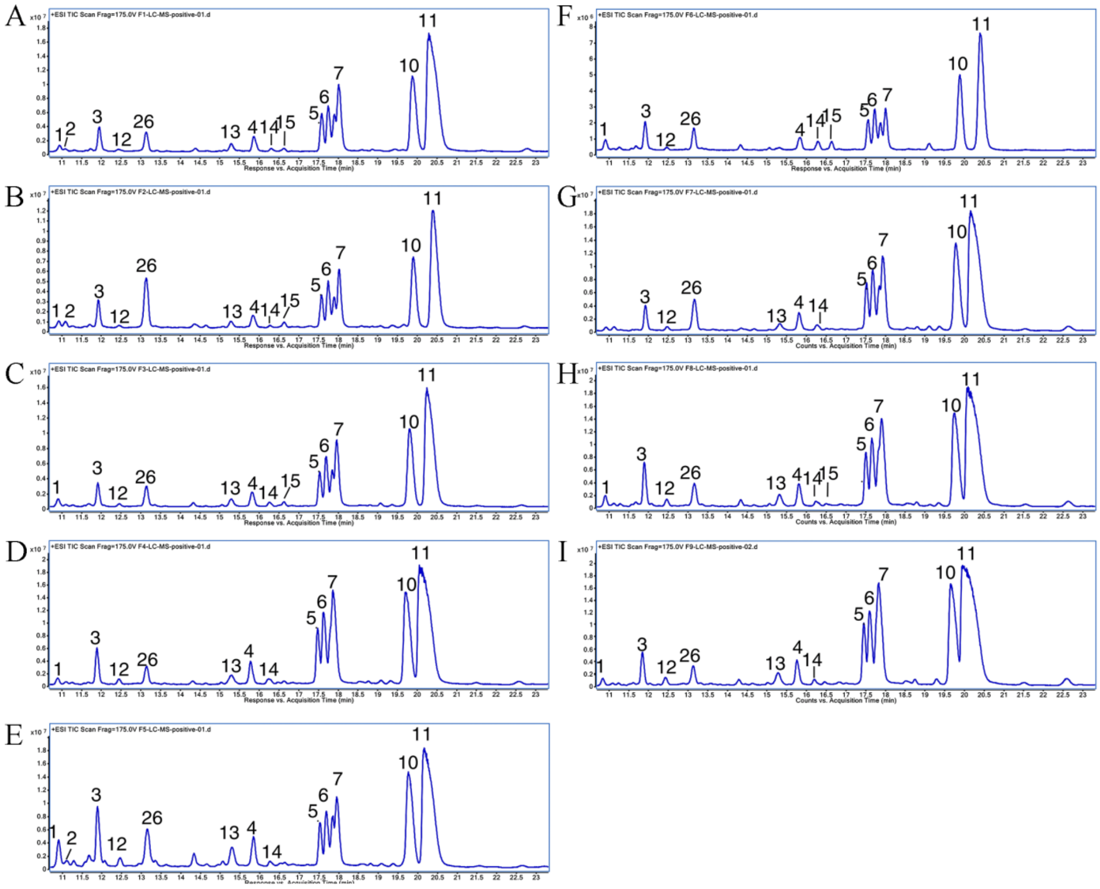

Figure S2 TIC chromatograms (11-23 min) of F1-F9 extracts in positive mode

Total ion current (TIC) chromatograms (11-23 min) of F1-F9 extracts analyzed by HPLC-QTOF-MS in a positive ion mode (A-I for F1-F9, respectively).

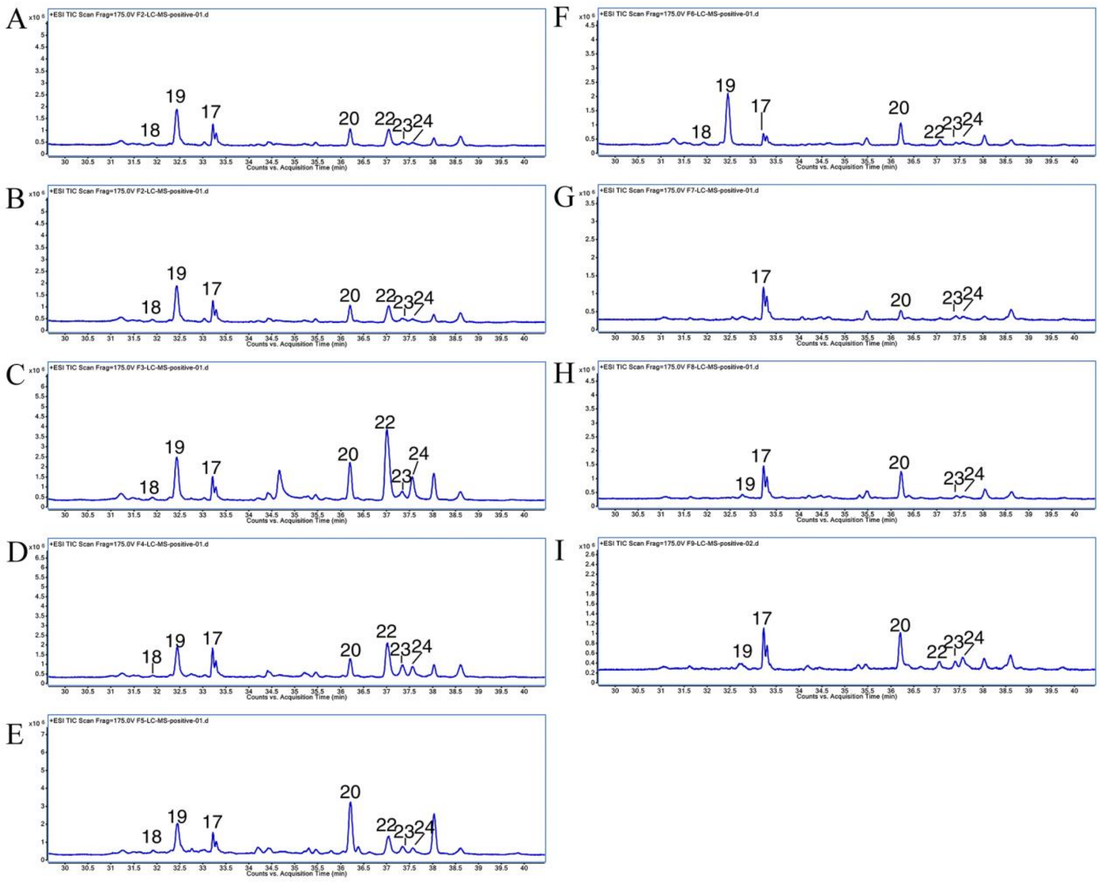

Figure S3 TIC chromatograms (30-40 min) of F1-F9 extracts in positive mode

Total ion current (TIC) chromatograms (30-40 min) of F1-F9 extracts analyzed by HPLC-QTOF-MS in a positive ion mode (A-I for F1-F9, respectively).

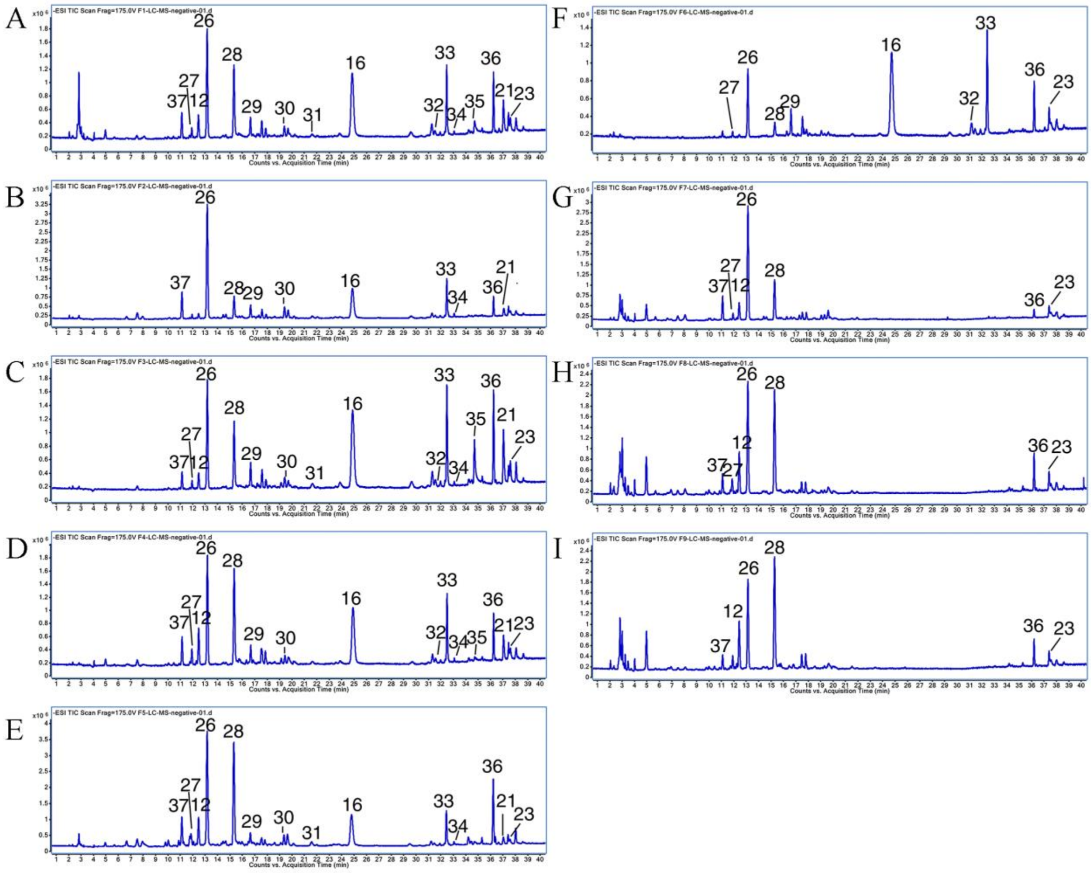

Figure S4 TIC chromatograms (1-40 min) of F1-F9 extracts in negative mode

Total ion current (TIC) chromatograms (1-40 min) of F1-F9 extracts analyzed by HPLC-QTOF-MS in a negative ion mode (A-I for F1-F9, respectively).

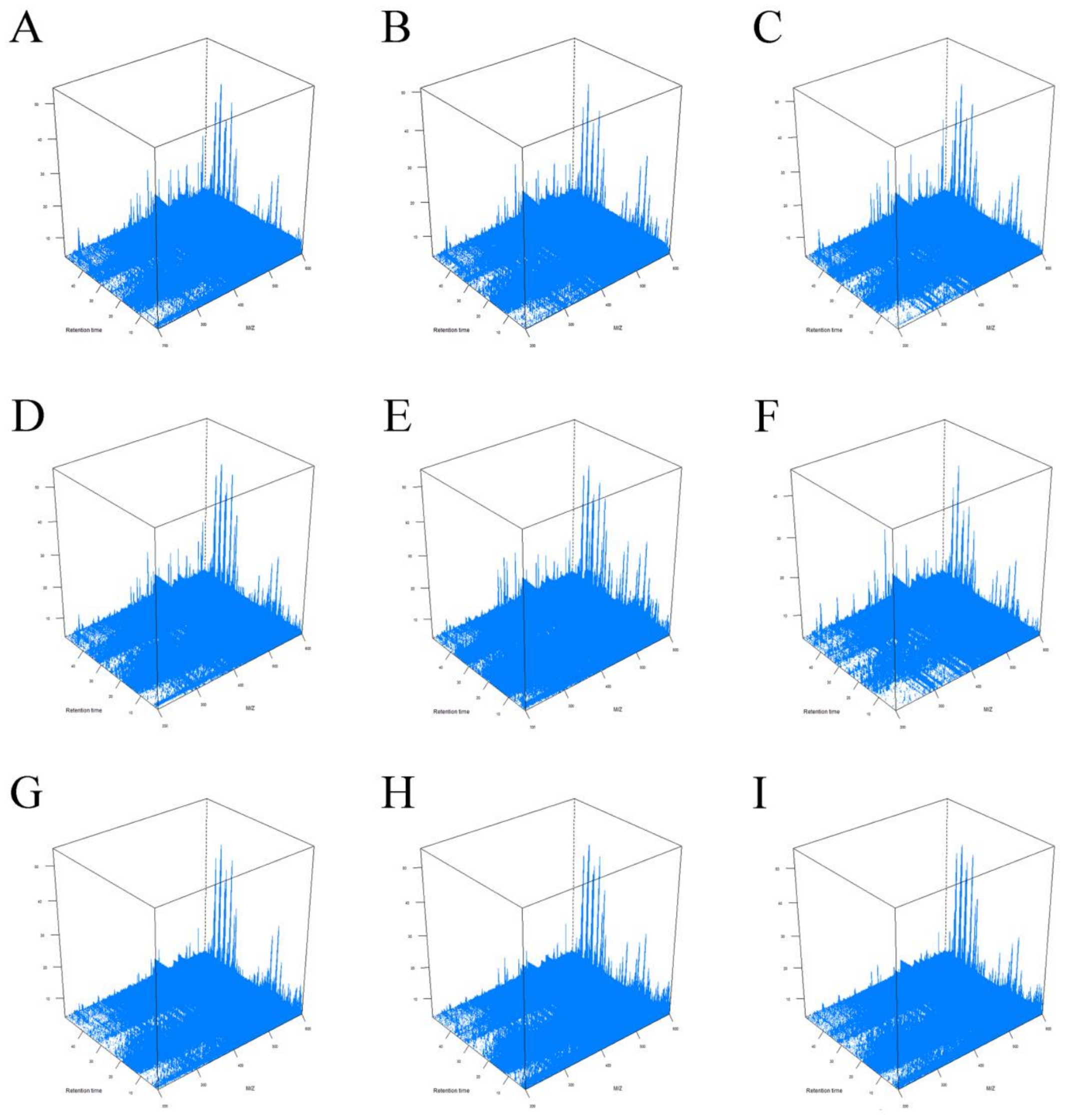

Figure S5 The 3D-TIC chromatograms of F1-F9 extracts in positive ion mode

The 3D total ion current (TIC) chromatograms of F1-F9 extracts analyzed by HPLC-QTOF-MS in a positive ion mode (A-I for F1-F9, respectively).

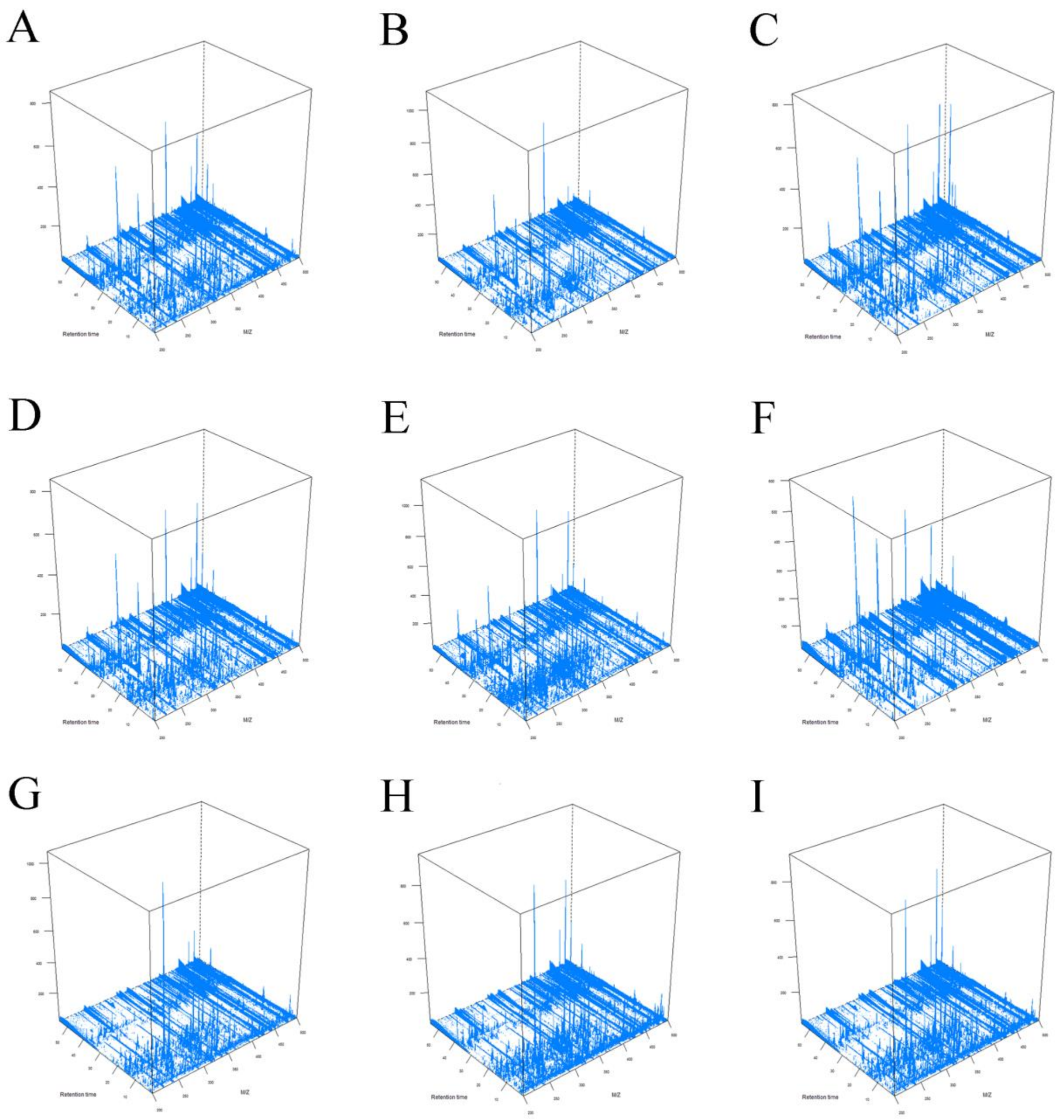

Figure S6 The 3D-TIC chromatograms of F1-F9 extracts in negative ion mode

The 3D total ion current (TIC) chromatograms of F1-F9 extracts analyzed by HPLC-QTOF-MS in a negative ion mode (A-I for F1-F9, respectively).

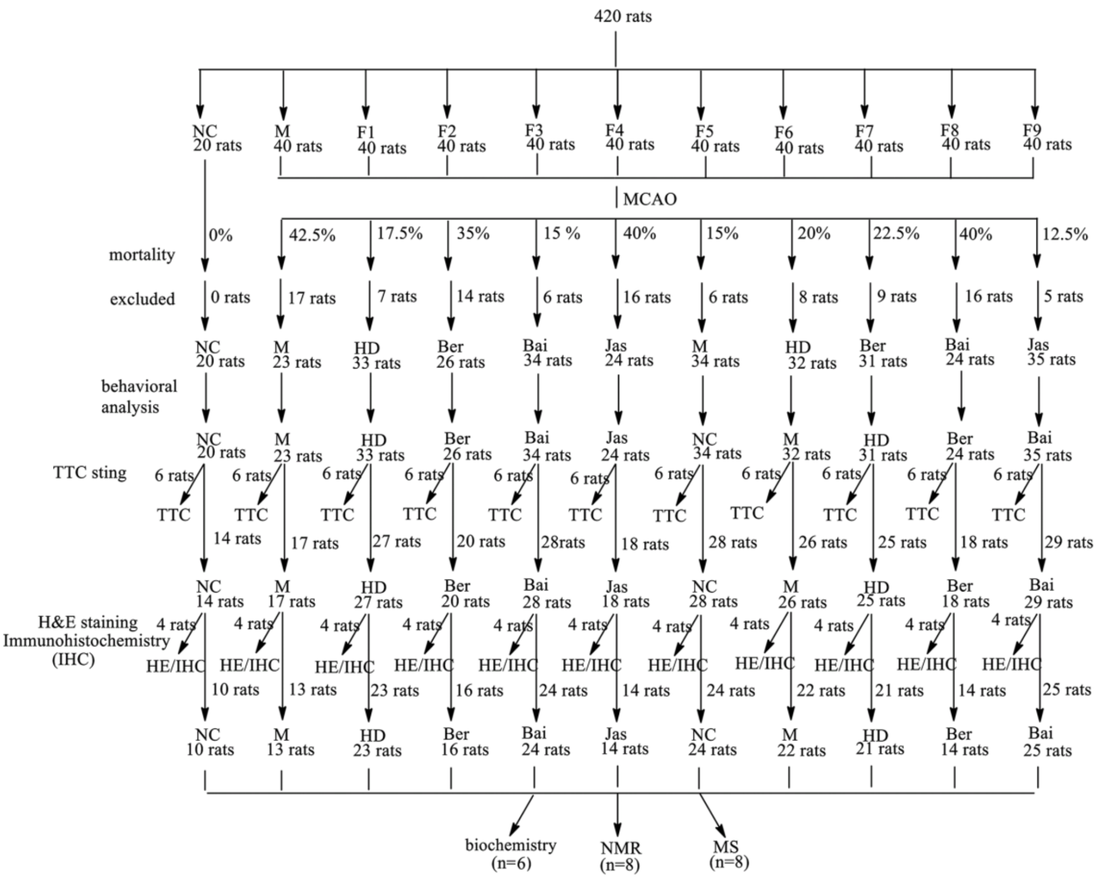

Figure S7 The flow chart diagram of the present experimental procedure.

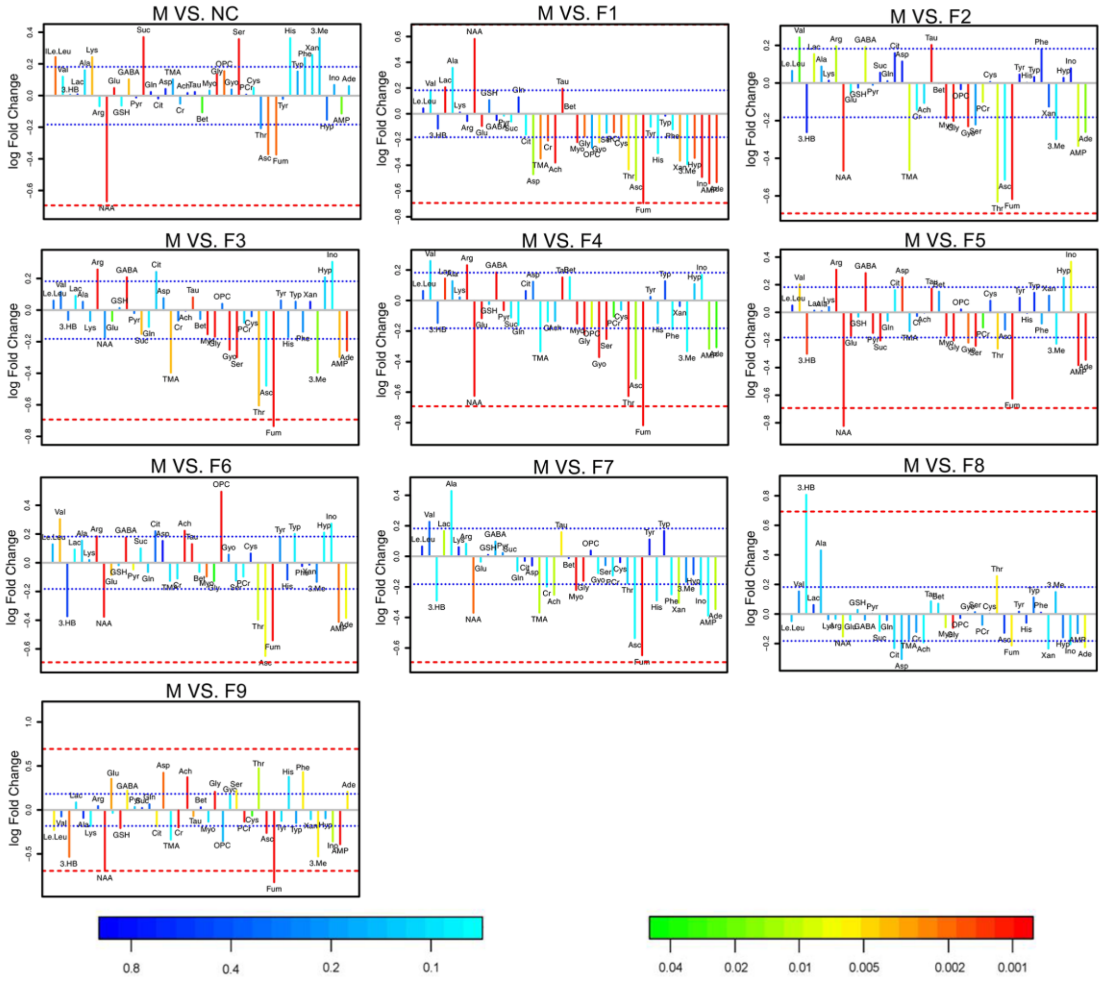

Figure S8 Fold change plots color-coded with *p*-values adjusted by Benjamini-Hochberg method

Color-coded fold change plots with *p*-values adjusted by BH method indicating significance of altered metabolites in brain extracts of each treated group versus MCAO group. The blue and red dashed lines represented variations of 20% and 100%, respectively.

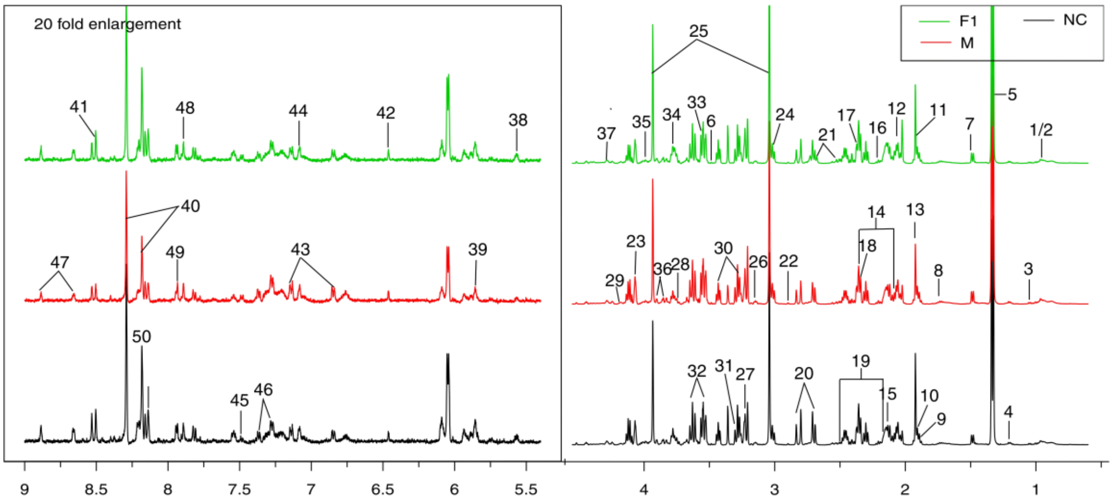

Figure S9 Typical 500 MHz ^{1}H NMR spectra

Typical 500 MHz ^{1}H NMR spectra of cerebrum obtained from the sham, the MCAO and the F1-treated groups. Metabolites in cerebrum tissue: 1, Isoleucine (Ile); 2, Leucine (Leu); 3, Valine (Val); 4, β-Hydroxybutyrate (3-HB); 5, Threonine (Thr); 6, Lactate (Lac); 7, Alanine (Ala); 8, Lysine (Lys); 9, Arginine (Arg); 10, γ-amino-butyrate (GABA); 11, Acetate (AC); 12, N-acetyl-aspartic acid (NAA); 13, N-acetyl-glutamate (NAG); 14, Methionine (Met); 15, Glutamate (Glu); 16, Glutathione (GSH); 17, Pyruvate (Pyr); 18, Succinate (Suc); 19, Glutamine (Gln); 20, Asparate (Asp); 21, Citrate (Cit); 22, Trimethylamine (TMA); 23, Creatinine (Cre); 24, Creatine (Cr); 25, Phosphocreatine (PCr); 26, Ethanolamine (ETA); 27, Choline (Cho); 28, Acetylcholine (Ach); 29, O-phosphocholine (OPC); 30, Taurine (Tau); 31, Betaine (Bet); 32, Myo-inositol (Myo); 33, Glycine (Gly); 34, Glycerol (Gyo); 35, Ascorbate (Asc); 36, Serine (Ser); 37, Inosine (Ino); 38, Uracil (Ura); 39, Uridine (UDP); 40, Adenosine (Ade); 41, Adenosine monophosphate (AMP); 42, Fumarate (Fum); 43, Tyrosine (Tyr); 44, Histidine (His); 45, Tryptophan (Trp); 46, Phenylalanine (Phe); 47, Nicotinamide (Nict); 48, Xanthine (Xan); 49, 3-Methylxanthine (3-MX); 50, Hypoxanthine (Hyp).

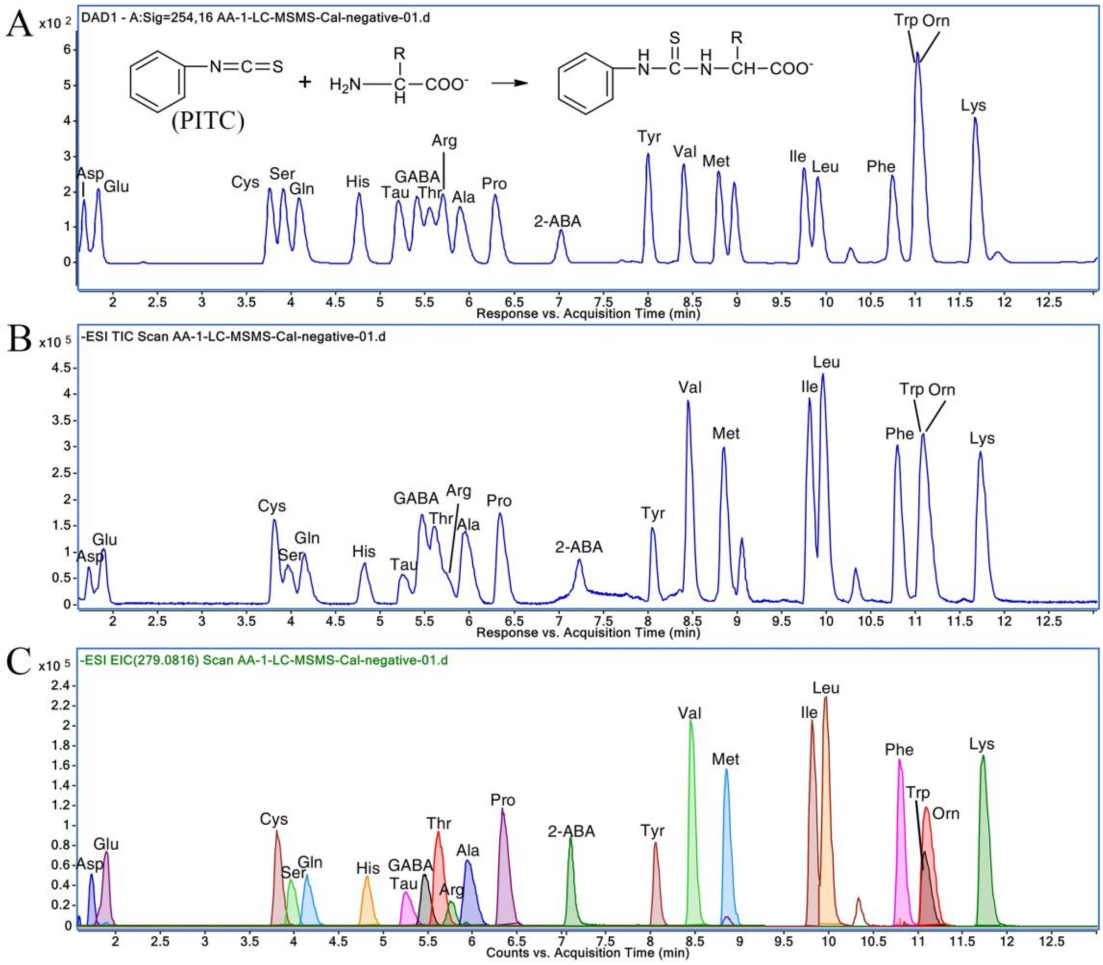

Figure S10 Profile of UV, TIC and EIC chromatograms of amino acids

(A) The UV chromatogram, **(B)** the total ion chromatogram and **(C)** the extract ion chromatogram of a mixture of amino acid standards solution.

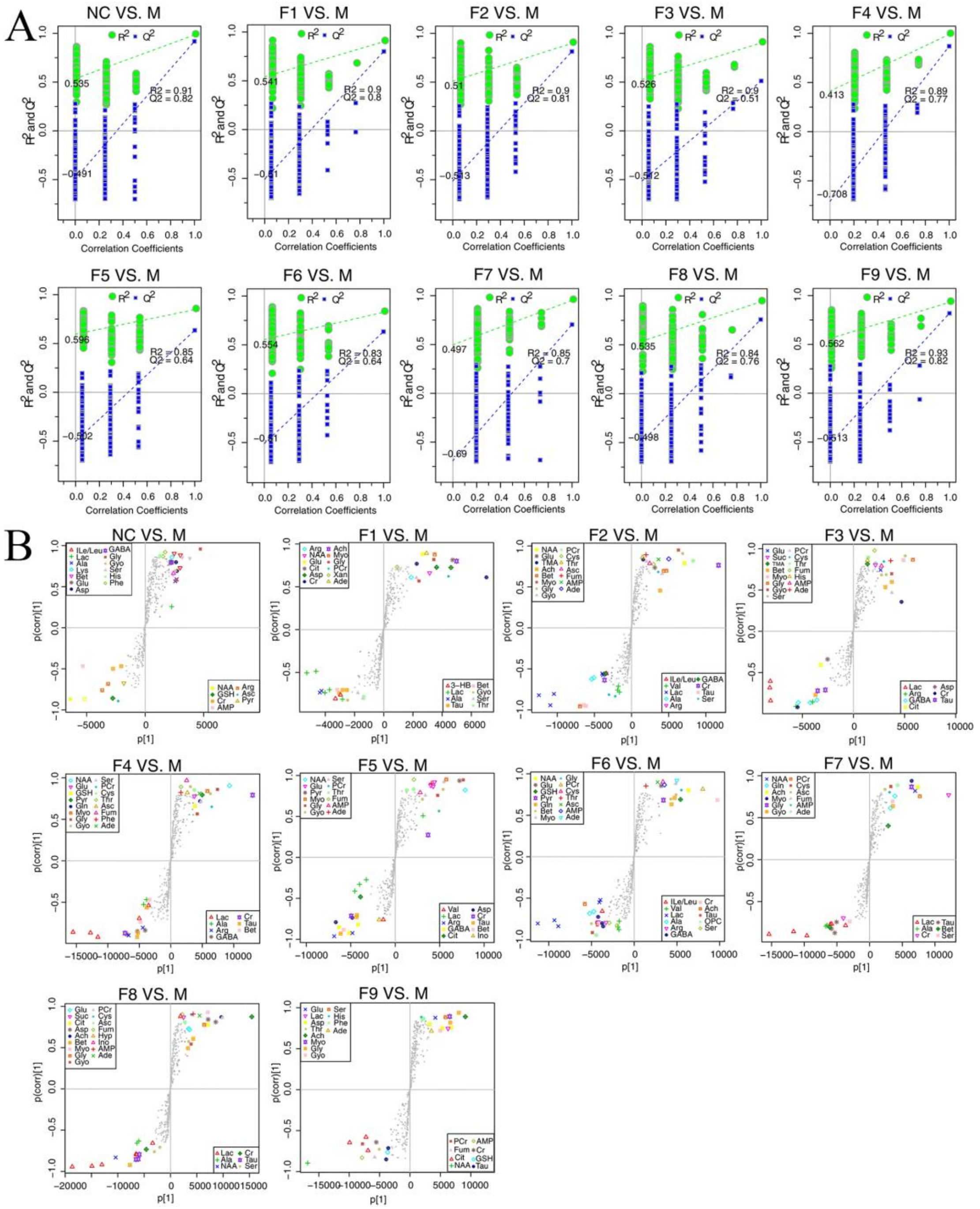

Figure S11 Scatter plots and S-plots for OPLS-DA analysis based on ^{1}H NMR spectra

(A) OPLS-DA scatter plots from cerebrum tissues of the statistical validations obtained by 2000 times permutation tests, with R^2 and Q^2 values in the vertical axis, the correlation coefficients (between the permuted and true class) in the horizontal axis, and OLS line representing the regression of R^2 and Q^2 on the correlation coefficients. **(B)** S-plots for OPLS-DA analysis of ^{1}H NMR data in cerebrum tissues.

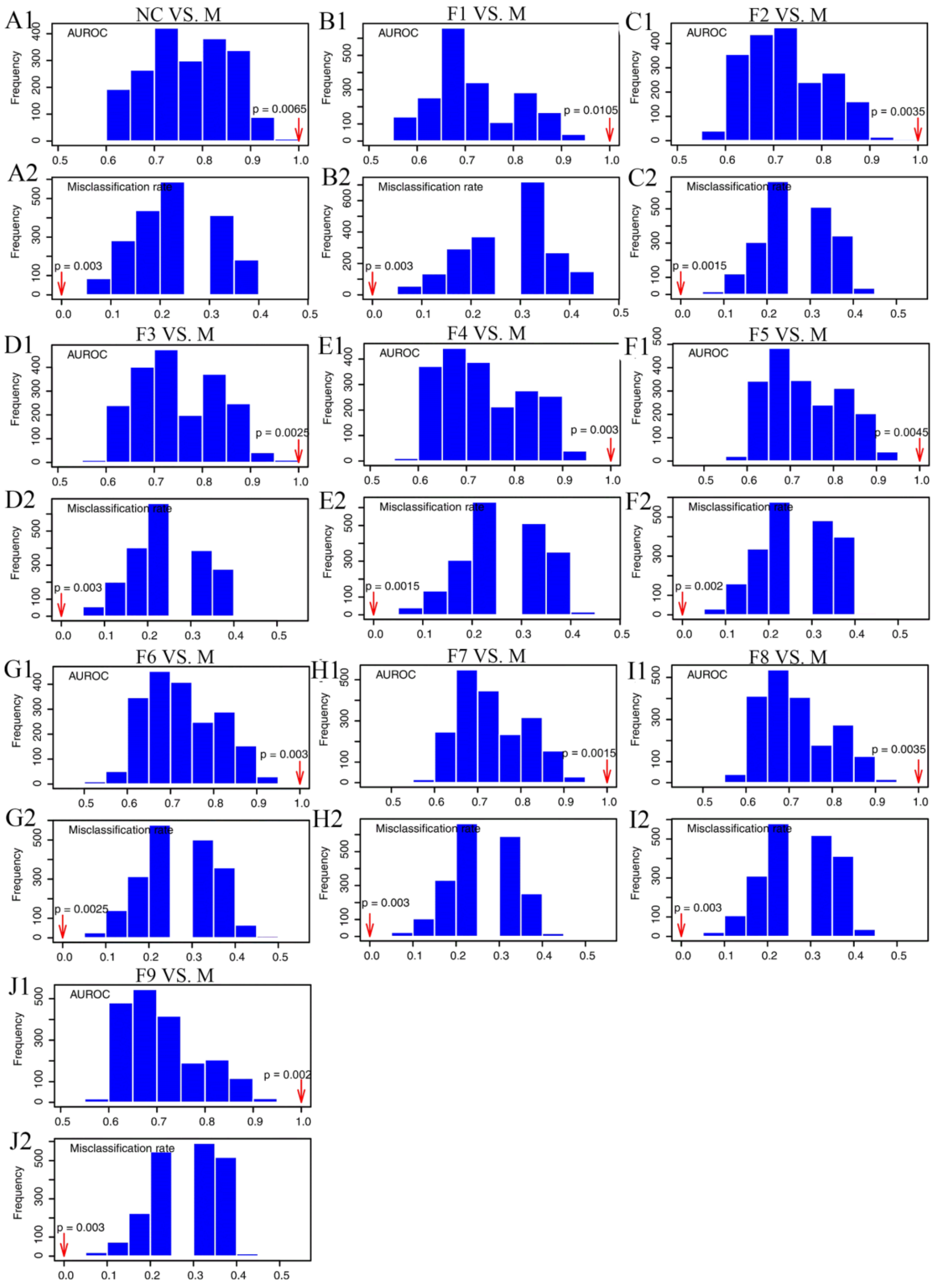

Figure S12 Histograms for **AUROC and the misclassification rate**

Histograms for class prediction results based on a 2000 times permutation test assessed AUROC (A1-J1) and the misclassification rate (A2-J2): the red arrow indicating the performance based on the original labels, a *p*-value less than 0.05 suggesting statistically significant separation of classes in the model.

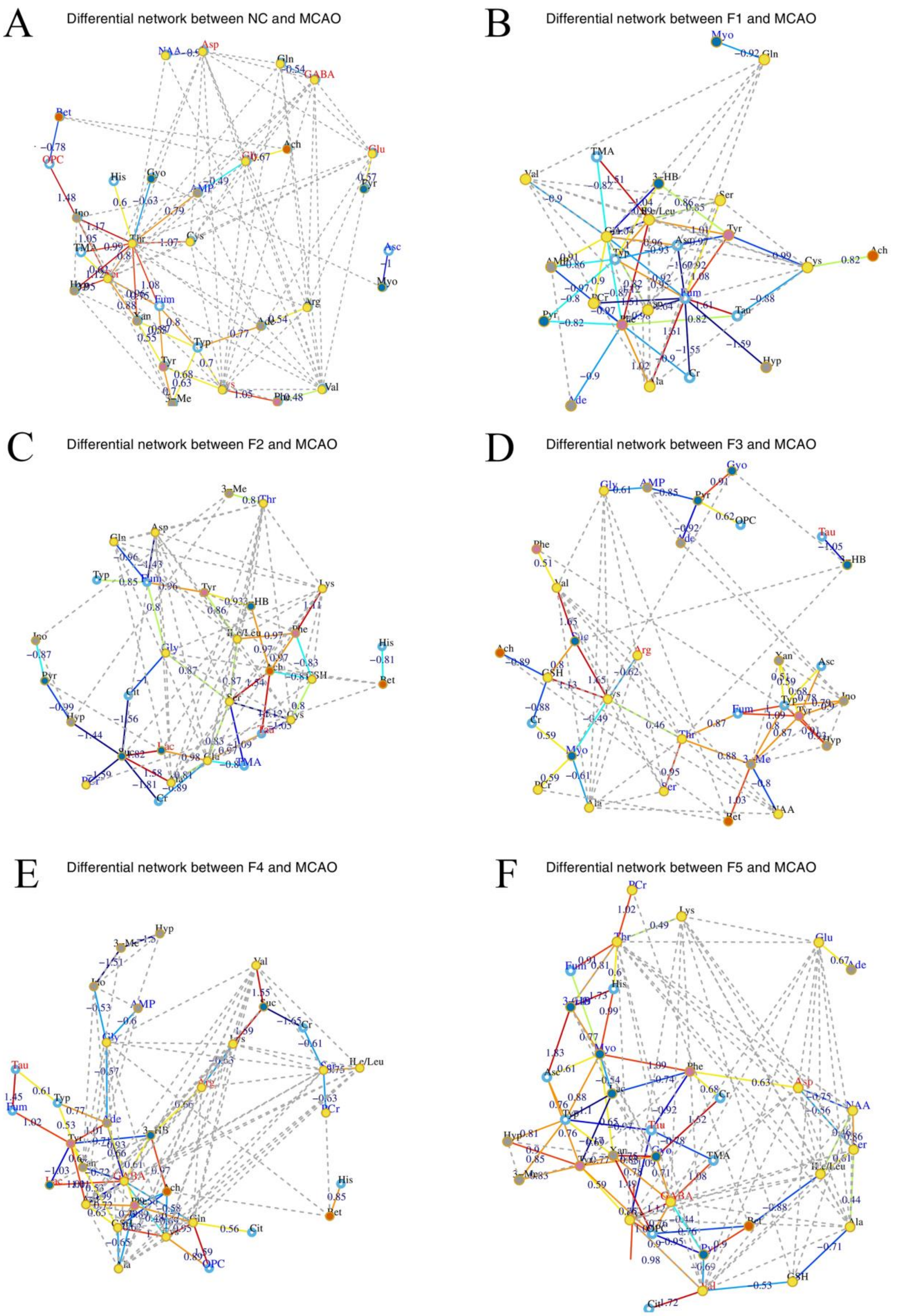

Figure S13-1 The differential networks of metabolites identified in global metabolomics

The differential networks were constructed based on the metabolites in brain tissues of sham and F1-9 groups, compared to MCAO rats. **(A)** Differential network between

NC and MCAO; **(B)** Differential network between F1 and MCAO; **(C)** Differential network between F2 and MCAO; **(D)** Differential network between F3 and MCAO; **(E)** Differential network between F4 and MCAO; **(F)** Differential network between F5 and MCAO.

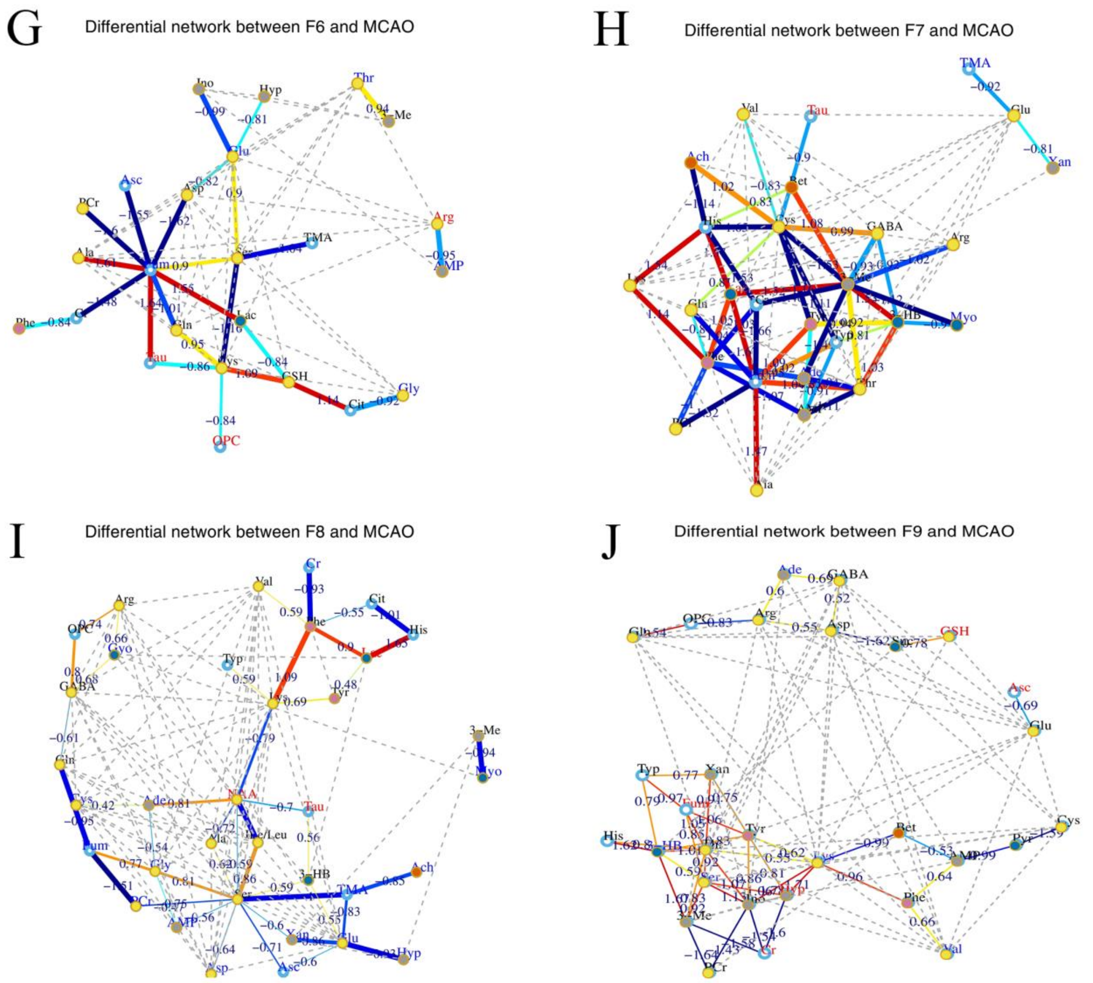

Figure S13-2 The differential networks of metabolites identified in global metabolomics

The differential networks were constructed based on the metabolites in brain tissues of sham and F1-9 groups, compared to MCAO rats. **(G)** Differential network between F6 and MCAO; **(H)** Differential network between F7 and MCAO; **(I)** Differential network between F8 and MCAO; **(J)** Differential network between F9 and MCAO.

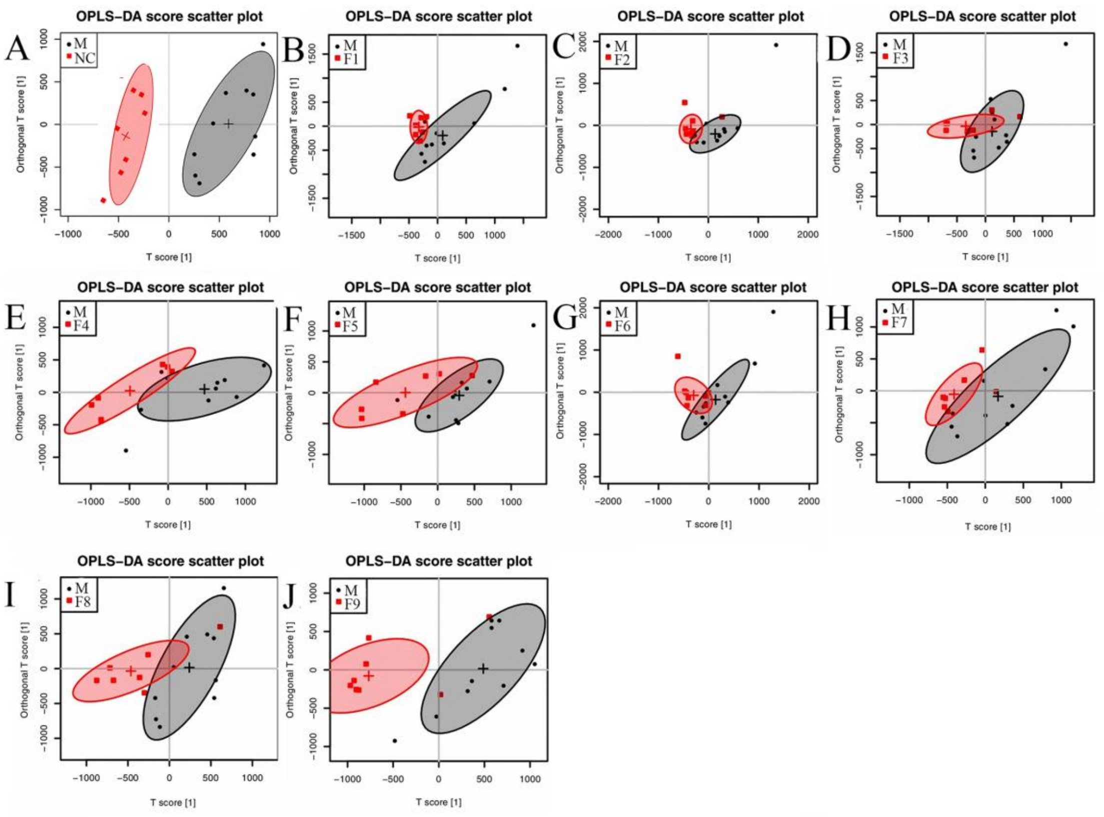

Figure S14 OPLS-DA score plots for amino acids

Score plots for OPLS-DA analysis based on amino acids targeted metabolomics analysis of cerebrum extracts obtained from the sham, the MCAO and all treated rats. **(A)** NC group VS. MCAO group; **(B-J)** Drug-treated groups VS. MCAO group. **B-J** for F1-F9, respectively.

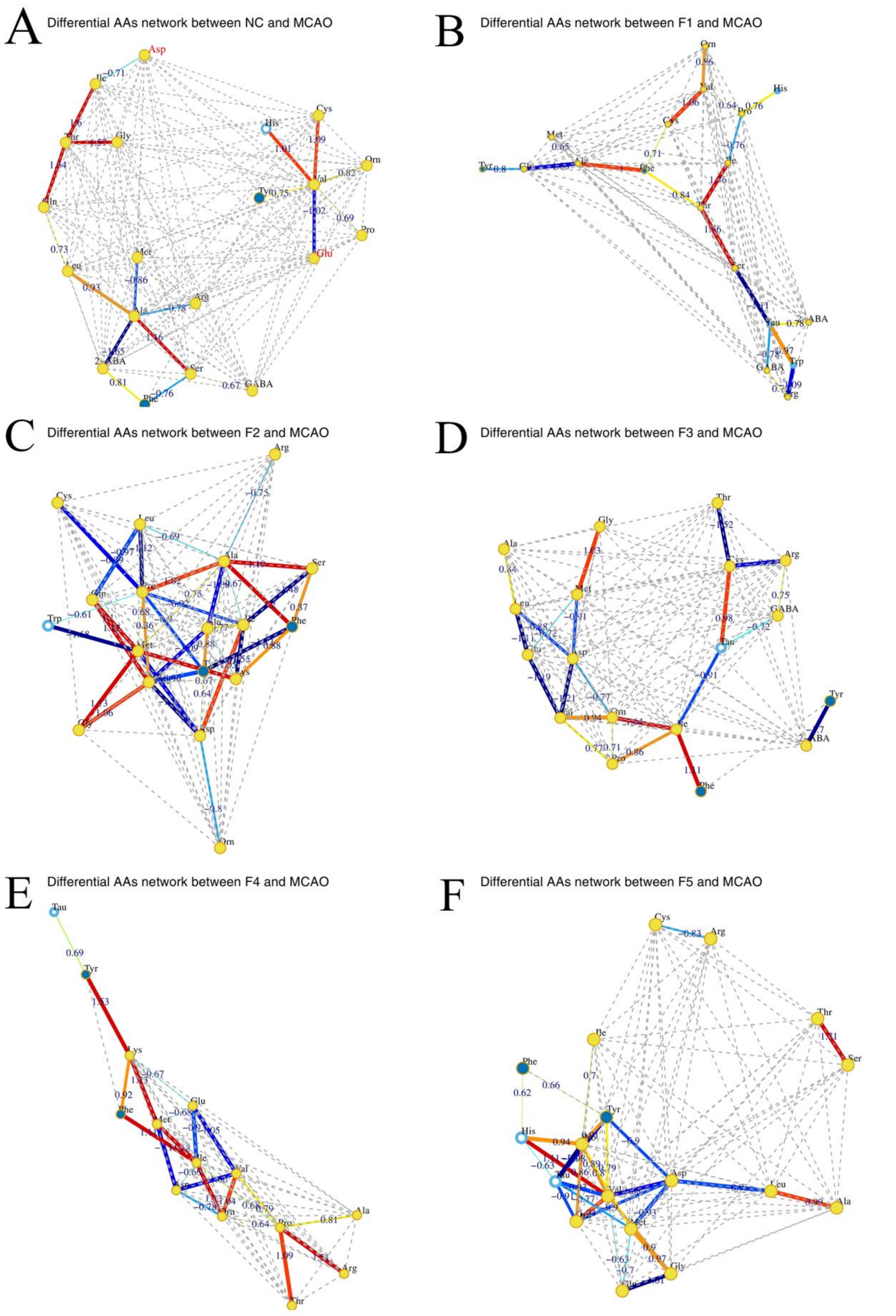

Figure S15-1 The differential networks of amino acids

The differential networks of AAs were constructed based on the differential amino acids in brain tissues of sham and F1-9 groups, compared to MCAO rats. **(A)** Differential network of AAs between NC and MCAO; **(B)** Differential network of AAs between F1 and MCAO; **(C)** Differential network of AAs between F2 and

MCAO; **(D)** Differential network of AAs between F3 and MCAO; **(E)** Differential network of AAs between F4 and MCAO; **(F)** Differential network of AAs between F5 and MCAO.

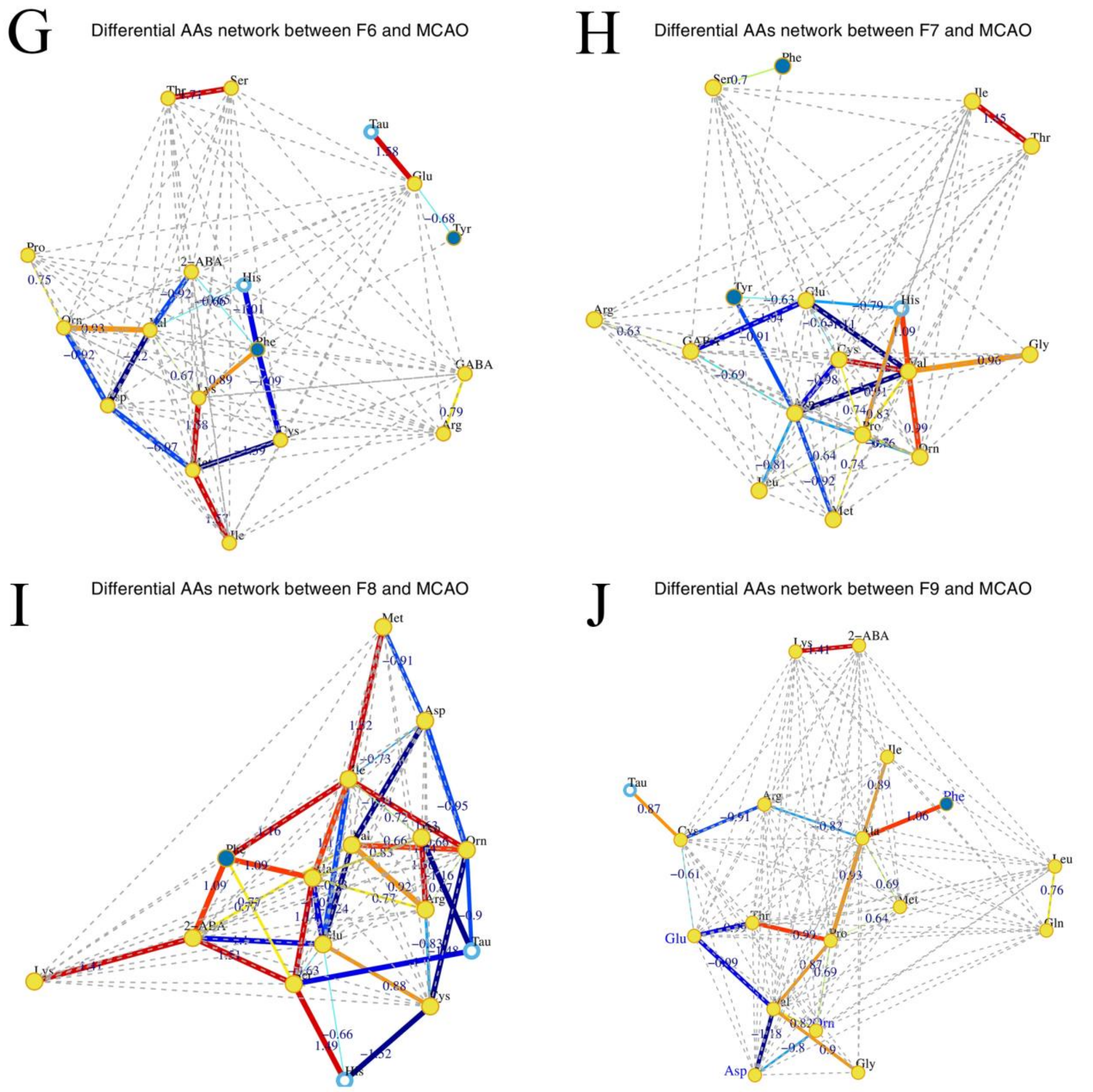

Figure S15-2 The differential networks of amino acids

The differential networks of AAs were constructed based on the differential amino acids in brain tissues of sham and F1-9 groups, compared to MCAO rats. **(G)** Differential network of AAs between F6 and MCAO; **(H)** Differential network of AAs between F7 and MCAO; **(I)** Differential network of AAs between F8 and MCAO; **(J)** Differential network of AAs between F9 and MCAO.
